# Structural organization of TAM receptor ectodomains

**DOI:** 10.1042/BCJ20260584

**Published:** 2026-10-08

**Authors:** Chrystal A. Starbird, Yuhong Zuo, Kahlil Walker, Steven E. Stayrook, Kathryn M. Ferguson

**Affiliations:** 1 Department of Pharmacology, Yale University School of Medicine, New Haven, CT 06520, U.S.A.; 2 Cancer Biology Institute, Yale University, West Haven, CT 06516, U.S.A.

**Keywords:** crystallography, ectodomain, receptor tyrosine kinases, small-angle scattering, TAM receptors, transmembrane proteins

## Abstract

TYRO3, AXL, and MERTK, the TAM receptor tyrosine kinases (RTKs), play roles in phagocytosis and negative regulation of immune responses, contributing to homeostasis in many adult tissues. Aberrant TAM signaling leads to diseases including autoimmune disorders, viral infection, inflammation, and cancer. Development of successful drugs targeting TAM receptors is hindered by incomplete knowledge of the receptor activation mechanism. Receptor dimerization is assumed to play a role, as seen for most RTKs. However, full biological responses require the TAM ligands to engage phosphatidylserine in an opposing cell or virus membrane, suggesting a more complex mechanism, possibly involving higher-order receptor oligomerization or clustering. Here, we present an X-ray crystal structure of the ectodomain of MERTK, which adopts a highly curved conformation with key interdomain boundaries stabilized by N-linked glycan moieties. We show that this MERTK extracellular region (ECR) forms a similar conformation in solution using small-angle X-ray scattering (SAXS), which also suggests a flexible ECR. We compare these crystallographic and solution data for MERTK with SAXS and AlphaFold structural predictions for the other two TAM receptors. SAXS data confirm that each TAM ECR has a distinct curvature, with TYRO3 the most bent and AXL relatively straight. This leads us to speculate that these different ectodomain conformations guide receptor-specific functions. The MERTK X-ray crystal structure also suggests a head-to-tail intermolecular interaction between the Ig2 domain of one MERTK and the FNIIIb domain of a second that has intriguing parallels with interactions that have been proposed to regulate Eph receptor clustering.

## Introduction

The TAM family of receptor tyrosine kinases (RTKs), comprising TYRO3, AXL, and MERTK, are expressed widely in the mature immune, vascular, nervous, and reproductive systems, where they act as key regulators of immune and vascular homeostasis and of inflammation [1,2]. Dysregulation of TAM receptors is implicated in numerous disease states, including autoimmune disorders, chronic inflammation, cancer, and cardiovascular disease [3–6]. In addition, TAM receptors can be exploited by viruses to facilitate infection of host cells [7–9]. TAM receptors suppress innate immune responses [1], in turn reducing activation and recruitment of T cells. TAM receptor inhibition can therefore modify the tumor microenvironment to promote the effectiveness of adaptive immune checkpoint inhibitors such as anti-PD1 [10]. However, MERTK also appears to play a co-stimulatory role in T cells [11], emphasizing the need for a more complete mechanistic understanding of this family of RTKs.

The mechanism of TAM receptor activation is incompletely understood. It is clear that simple binding of the TAM ligands (growth arrest-specific 6/GAS6 and Protein S/PROS1) to their receptors is not sufficient to induce full biological responses [4,12–15]. Engagement of phosphatidylserine (PtdSer) displayed on an opposing cell (or virus) membrane—through the ligand’s N-terminal γ-carboxyglutamate-rich (Gla) domain [14,15]—strongly promotes TAM receptor activation. Mounting evidence suggests that PtdSer-dependent organization or clustering of TAM receptors may contribute to their regulation [16,17]. Thus, TAM receptor activation appears to involve extracellular interactions beyond simple ligand-induced dimerization. Higher-order receptor organization is increasingly recognized as an important regulatory feature of several other RTK families, including the TIE (tyrosine kinase and immunoglobulin-like and epidermal growth factor homology domains) and Eph (erythropoietin-producing human hepatocellular) receptor families [18,19]. Interestingly, these receptors share certain features in their extracellular regions (ECRs) in common with TAM receptors, notably having membrane-proximal fibronectin type III (FNIII) domains. In TIE2 and Eph receptors, the FNIII domains have been suggested to mediate ligand-independent receptor interactions that modulate receptor regulation [20–25]. Whether the TAM receptor FNIII domains participate in analogous interactions is unknown.

Mouse genetic studies have been instrumental in dissecting the biological roles of TAM receptors [26–28] and reveal substantial functional redundancy among the members of this RTK family [13,28], although each receptor has a distinct expression pattern [29]. Together with functional studies, these findings have established important roles for TAM receptors in processes including phagocytosis and negative regulation of immune responses [1,2,28]. Nevertheless, the three receptors are not functionally equivalent, raising the possibility that differences intrinsic to the receptors contribute to their distinct biological activities [14,30,31]. One well-established distinction lies in ligand recognition: AXL binds GAS6 with high affinity [32] and, in most settings, is not activated by PROS1 [13,14,33,34], whereas TYRO3 and MERTK bind GAS6 with lower affinity [32] and can also be activated by PROS1 [14]. These differences are generally considered in terms of ligand specificity and cellular context, but much less is known about whether the extracellular architecture of the receptors themselves differs across the TAM family. The ECRs of all three receptors share the same domain organization, comprising two N-terminal immunoglobulin-like domains followed by two membrane-proximal FNIII domains. It is not known how these domains are arranged or whether receptor-specific ECR conformations might contribute to differences in receptor organization and regulation.

Here, we present an X-ray crystal structure of the MERTK ECR that shows an extended, curved structure, with interdomain orientations that appear to be stabilized by N-linked glycans. Small-angle X-ray scattering (SAXS) data indicate that the solution conformation of MERTK is consistent with the crystal structure while also indicating interdomain flexibility. Comparison with SAXS data for TYRO3 and AXL supports distinctly different ECR curvatures among the three TAM receptors, consistent with differences predicted by AlphaFold2 (AF2) models [35]. These observations suggest that, despite sharing the same domain organization, TAM receptor ECRs have distinct conformational properties that may constrain the geometries available for receptor assemblies. The MERTK crystal structure additionally uncovers intermolecular contacts resembling interactions seen in the Eph receptor family [19,23,36,37], raising the possibility that related modes of extracellular receptor–receptor interactions contribute to the regulation of MERTK and potentially other TAM receptors.

## Results

### The X-ray crystal structure of MERTK ECR shows an elongated, curved structure

The purified ECR of MERTK (sMERTK), secreted from Sf9 cells using the native signal sequence and with a His_6_ tag following amino acid 484, was crystallized with PEG 3350 as precipitant at pH 8.0. Crystals belonged to space group P2_1_2_1_2_1_ and diffracted to 3.4 Å resolution. Data were collected at the Advanced Photon Source (Argonne National Laboratory), and the structure was solved using molecular replacement (MR) methods with search models generated in Phyre2 [38] and/or AF2 [35]. There is one molecule in the asymmetric unit and 62%–66% solvent (depending on the exact extent of glycosylation). Details of data collection and refinement are in Supplementary Table S1.

The sMERTK, which comprises tandem Ig domains followed by tandem FNIII domains (Figure 1A), adopts an extended structure with pronounced curvature (Figure 1B). The successive bends between domains (42°, 31°, and 67° between Ig1/Ig2, Ig2/FNIIIa, and FNIIIa/FNIIIb, respectively) result in the long axis of the structure following an arc of ∼145° around a circle of diameter ∼120 Å. Searches using the DALI server [39] identify the olfactory cell adhesion molecule NCAM2 as a structural homologue of MERTK. Indeed, this is the only structure outside the TAM receptor family with a similar overall curvature in searches of both experimentally determined and AlphaFold-predicted structures. An X-ray crystal structure of an NCAM2 fragment comprising the membrane-proximal two Ig domains plus two FNIII domains [40] has a remarkably similar overall curvature to that seen in the MERTK crystal structure. For NCAM2, this curvature is proposed to facilitate the *trans*-interactions of the membrane distal Ig1 domains of NCAM2 molecules across two closely opposing cell membranes, such as at a synapse [40,41]. Interestingly, MERTK was designated an NCAM-related tyrosine kinase by one of the groups that cloned the human gene in the early 1990s [42]. Moreover, structural studies of the TYRO3 Ig1–Ig2 module provided evidence for homotypic interactions for this TAM receptor [43], suggesting potential direct participation of TAM receptors in cell adhesion. It is, however, more generally accepted that the role of TAM receptors in cell-cell contacts is dependent on ligand and/or mediated by indirect signaling events [1,13,44]. Nevertheless, the curvature of MERTK may play a role in defining the geometry of functional receptor assemblies, as we discuss in more detail below.

**Figure 1:**
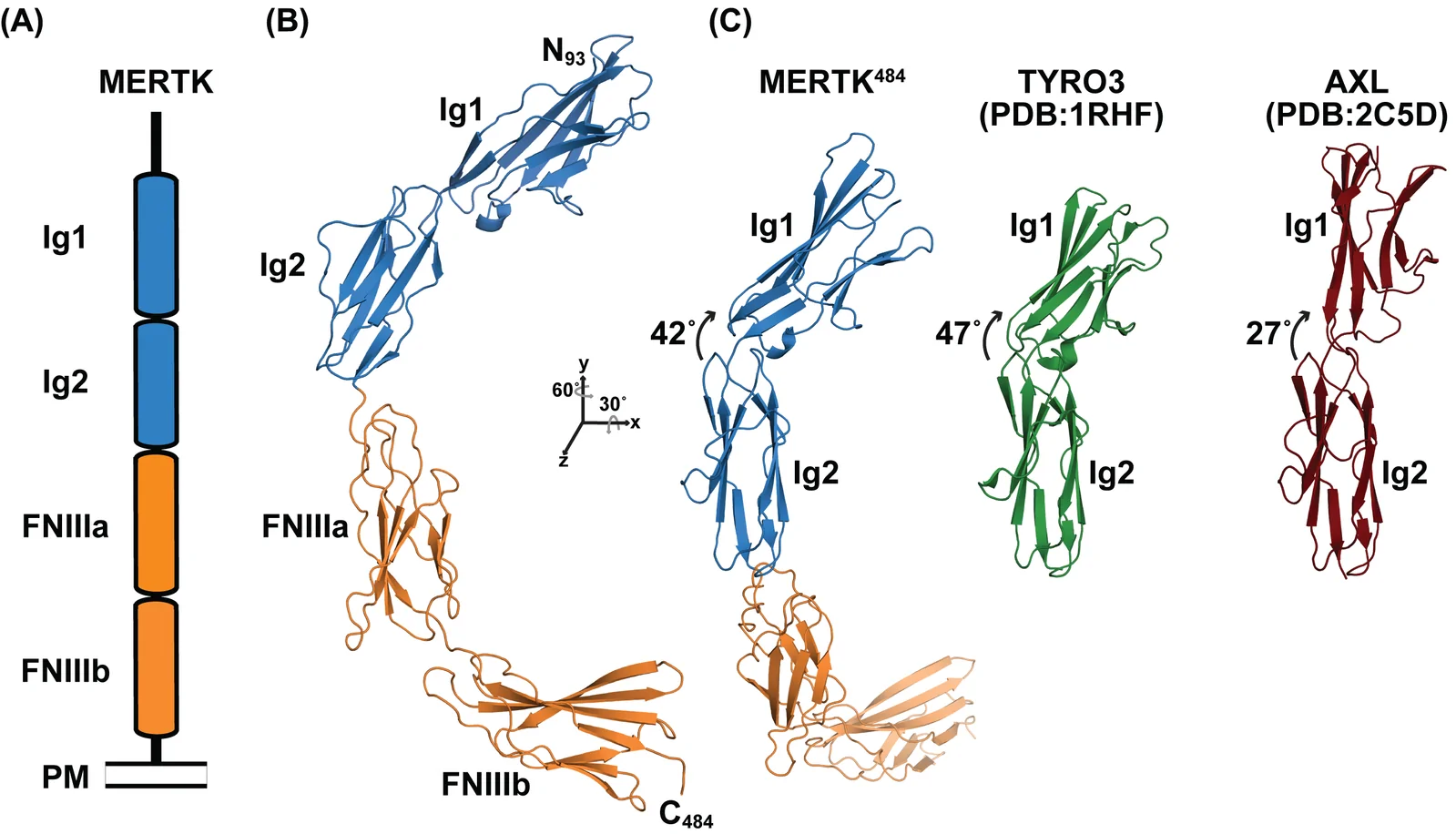
The MERTK structure has an extended curved conformation (**A**) The domain architecture of the MERTK ECR is shown with the two Ig domains colored in blue and the two FNIII domains in orange. The location of the plasma membrane (PM) is indicated. (**B**) A cartoon view of the X-ray crystallographic model of the MERTK ECR (MERTK^484^) with domains colored as in panel (A). The amino acid numbers for the N- and C-termini of the model are indicated. The N-terminal ∼70 amino acids are disordered in the crystal. (**C**) Comparison of the Ig1–Ig2 module of MERTK^484^ with known crystal structures of this module from TYRO3 and AXL. The view is rotated 30° and 60° about the *x*- and *y*-axis, respectively, to highlight the Ig1–Ig2 interdomain relationship. For MERTK^484^ and TYRO3 (PDB: 1RHF in green) [43] the interdomain angles are similar (42° and 47°, respectively). By contrast, this module of AXL (PDB: 2C5D in dark red) [45] is less bent with an interdomain angle of 27°.

Although no structures have been reported for the MERTK ECR, X-ray crystal structures of the tandem Ig domains from both TYRO3 and AXL have been reported, the latter in complex with the receptor binding region of the ligand, GAS6 [43,45]. The bend between the two Ig domains of MERTK is similar to that observed for TYRO3 (42° and 47°, respectively), whereas for AXL the long axes for the two Ig domains are more aligned (27°; Figure 1C). Both Ig domains are of the I-set subclass. The first Ig domain has canonical I-set strand topology, as seen for AXL, but not TYRO3, which has non-canonical strand crossover between the two β-sheets [43]. The second Ig domain of MERTK shares significant sequence identity with those of other TAM receptors—∼40% in pairwise alignments compared with 30% for Ig1 alone and across the entire ECR (Supplementary Figure S1). Indeed, the Ig2 domains of TAM receptors form a distinct subclass of I-set Ig domains (cd05749: IgI_2_Axl_Tyro3_like) [46]. Consistent with this, the root mean square deviation (RMSD) for mainchain atoms of the Ig2 domain of MERTK is just 1.02 and 2.02 Å relative to TYRO3 (PDB: 1RHF) [43] and AXL (PDB: 2C5D) [45], respectively, compared with 4.12 and 2.57 Å for the Ig1 domains. The only experimentally determined structure of a TAM FNIII domain is a nuclear magnetic resonance structure of the second FNIII domain from MERTK (PDB: 2DBJ) [47], which overlays with this domain in our MERTK X-ray crystal structure with an RMSD of 1.3 Å.

MERTK crystallized with one molecule in the asymmetric unit. Interestingly, one of the crystal packing interactions involves the presumed ligand-binding region of the second Ig domain (Ig2), based on interaction seen in the X-ray crystal structure of the receptor binding region of GAS6 in complex with the two Ig domains of AXL [45] (Figure 2A,B). Beta-strand G of Ig2 forms a parallel interaction with the β-strand E of neighboring FNIIIb (FNIIIb′) in a manner that closely resembles the interaction of this same β-strand of the Axl Ig2 with the β-strand J of the first Laminin G-like (LG) domain (LG1) of GAS6 (Figure 2C). In both cases, the interaction is capped at each end of the β-strand by additional hydrogen bond interactions (indicated by dashed lines in Figure 2C). The surface areas occluded from solvent are also similar: 765 and 934 Å^2^ for the AXL Ig2/LG1 and MERTK Ig2/FNIIIb interactions, respectively. As discussed in detail below, we speculate that this heterotypic Ig/FNIII domain interaction may play a role in MERTK oligomerization and/or clustering.

**Figure 2:**
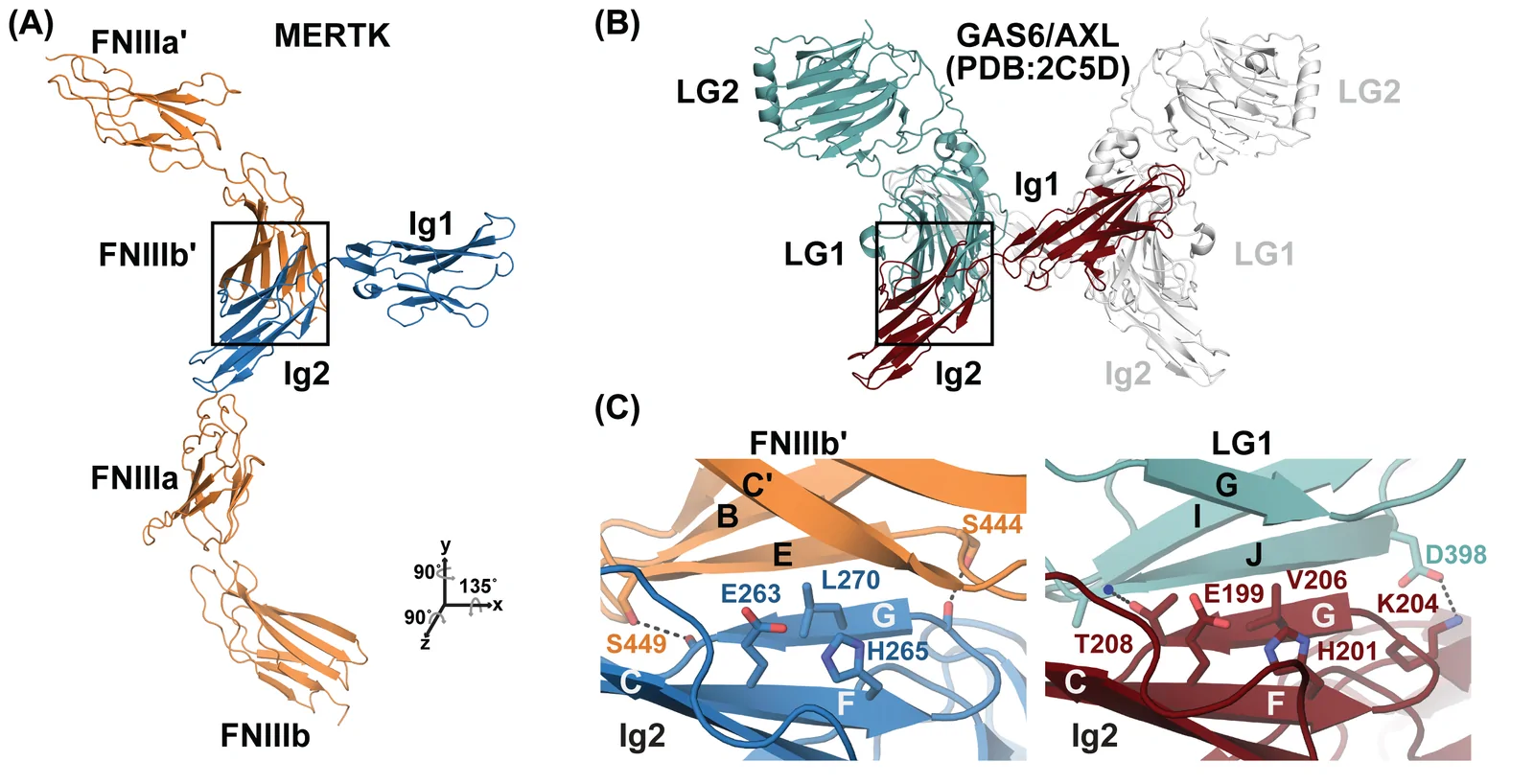
An intermolecular contact between an FNIIIb domain and the presumed ligand binding site on Ig2 (**A**) The MERTK^484^ structure is shown colored as in Figure 1A. The two FNIII domains of a crystallographically related MERTK^484^ molecule are shown in orange and labelled FNIIIa′ and FNIIIb′. FNIIIb′ interacts with Ig2 of the first MERTK^484^ molecule. The orientation is such that the Ig2 domain overlays with the Ig2 domain of the left-hand protomer of the GAS6/AXL complex shown in B. The box indicates the area of detail shown in panel (C). (**B**) Cartoon representation of the Ig1**–**Ig2 module of AXL bound to the LG1–LG2 or SHBG module of GAS6 (PDB: 2C5D) [45]. In the left-hand protomer, the AXL is in dark red and the GAS6 in teal. The right-hand protomer is white. Box shows the area of detail in panel (C). (**C**) A close-up view of the interactions with the Ig2 domains of MERTK and AXL colored as in panels (A) and (B). β-strands are labelled, and key side chains at the interface are shown in stick representation. On the left, the interaction of MERTK Ig2 with the FNIIIb′ from a symmetry mate is shown. Ig2 β-strand G forms a parallel interaction with β-strand E of FNIIIb′, and the interaction is capped at each end by presumed hydrogen bonds involving S444 and S449 from FNIIIb′. On the right, a similar view is shown for the GAS6-LG1/AXL–Ig2 interaction. Here Ig2 β-strand G interacts with β-strand J of the GAS6 LG1, and hydrogen bonds cap each end of this interaction. The rotations relating the orientation in panels (A) and (B) with that in panel (C) are indicated (135° about the *x*-axis, 90° about the *y*-axis, and 90° about the *z*-axis).

### The role of N-linked glycosylation in MERTK organization

The ECR of MERTK is highly glycosylated with up to 14 potential N-linked glycan sites [48], which is about 3.5 sites per 100 amino acids (or one every 28 amino acids), considering only the folded portion of the ECR (amino acids 92–484; Supplementary Figure S1). This is twice as many sites as predicted for AXL or TYRO3 (Figure 3A). Many RTKs are rich in N-linked glycosylation sites, which have been proposed to play both structural and functional roles [49–51]. This has been very well studied in, for example, the epidermal growth factor receptor, which is ∼30% sugar by mass, where glycosylation is essential for receptor maturation, folding, and function [52,53], with similar observations for the extensively glycosylated vascular endothelial growth factor receptor 2 [54]. Alterations in RTK glycosylation are frequently linked to diseases, especially cancer [55]. For MERTK, trafficking and folding defects that are induced following prolonged ligand exposure in leukemia are attributed to incompletely glycosylated receptors [56], and in liver cancer, MERTK is stabilized by glycosylation to promote tumor growth [57].

**Figure 3:**
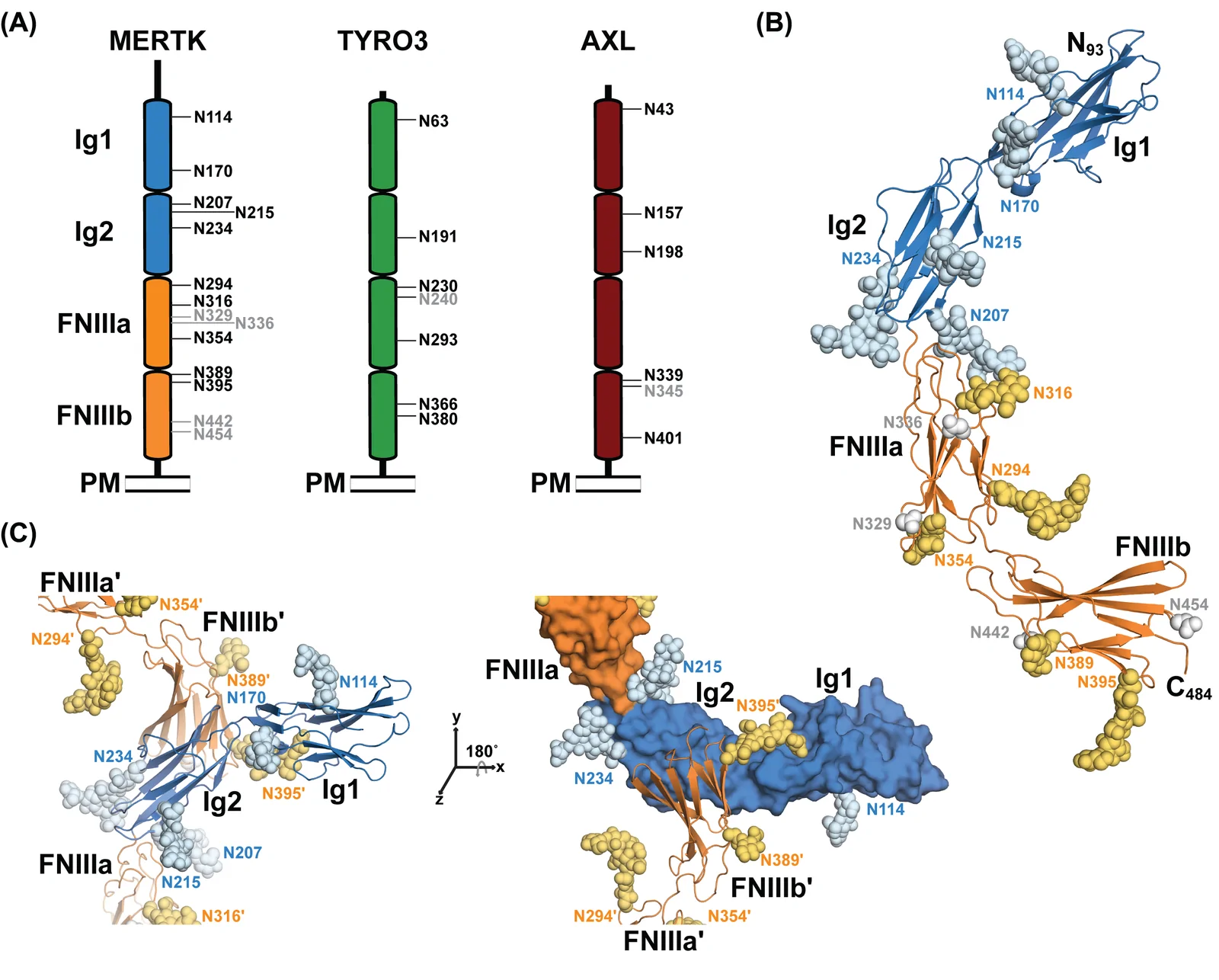
Roles for the N-linked glycans of MERTK (**A**) The locations of glycosylation sites on MERTK, TYRO3, and AXL are shown on domain architecture cartoons. MERTK cartoon is as in Figure 1A. For TYRO3, all domains are in green, and dark red for AXL. Sites where glycans are modeled in the MERTK X-ray crystal structure are indicated in black on the MERTK cartoon, whereas those where little or no density for sugar groups could be seen are in gray. Sites in TYRO3 and AXL with a low probability of being glycosylated [48] are also in gray (see the ‘Methods’ section). (**B**) MERTK^484^ is shown as in Figure 1B, with the modeled glycans shown as spheres in colors that are a lighter shade than the domain to which they are attached. Asparagine side chains at positions predicted to be glycosylated by the NetNGlyc-1.0 webserver [48] for which little or no density is seen in experimental maps are shown in white spheres. Note that several of the glycan chains are close to domain boundaries. (**C**) The interaction between Ig2 and FNIIIb′ shown in Figure 2C is augmented by interactions involving the glycan chain on N395′. The left-hand view is in the same orientation as Figure 2A, here focusing on the region of interaction. Upon a 180° rotation about the *x*-axis, the interaction of the glycan with Ig1 is apparent. In the right-hand view, the molecule with which FNIIIb′ interacts is shown in surface representation (excluding the glycans).

The MERTK used in the present study was expressed and secreted from Sf9 cells and therefore is expected to be decorated predominantly with high-mannose, or short mannose, glycans such as paucimannose [58]. We observe at least one sugar unit at 10 of the 14 predicted N-linked glycosylation sites in the electron density maps for our X-ray crystal structure (Supplementary Figure S2A,B). All predicted sites are glycosylated in Ig1 and Ig2 with 2–4 sugar residues modeled (Figure 3B). By contrast, we can model sugars at only five of eight predicted glycosylation sites in the two FNIII domains, two of which are poorly ordered with only one N-acetylglucosamine modeled.

Several of the modeled glycans in MERTK lie close to interdomain boundaries and may contribute to constraining and/or stabilizing the relative domain orientations (Figure 3B). For example, the glycan on N170 sits close to the Ig1/Ig2 boundary, and that at N207 is close to the Ig2/FNIIIa boundary. At the FNIIIa/FNIIIb boundary, the glycan on N294 makes a crystal packing contact with FNIIIb′ of a symmetry mate. In the absence of this interaction, this glycan may contribute to stabilization of the FNIIIa/FNIIIb domain junction. N-linked glycan chains were important in maintaining TRKA (tropomyosin receptor kinase A) in an extended conformation in a recent study using molecular dynamics simulations of TRKA, which shares a similar density of N-linked glycosylation sites to MERTK (1 per 26 amino acids) [59]. As for MERTK, the N-glycans for TRKA are concentrated at the domain junctions. We speculate that the N-linked glycosylation of MERTK plays a role in maintaining the extended, curved conformation observed in our X-ray crystal structure.

Intriguingly, the Ig2/FNIIIb′ interaction shown in Figure 2A,C that occludes the presumed ligand binding region of Ig2 is augmented by interaction of the glycan at N395′ on the FNIIIb′ with the Ig1 of the other molecule (Figure 3C and Supplementary Figure S2B,D). This interaction buries an additional 286 Å^2^ from solvent—23% of the total surface area occluded in the interaction of these two MERTK molecules. It is also of note that the glycan at N114, in the Ig1 β-strand B, is very close to the presumed ligand binding site on the Ig1 domain; indeed, the side chain at the analogous position in AXL, R48, makes direct interactions with GAS6. TYRO3 also has an N-linked glycosylation site in the middle of Ig1 β-strand B (on N63), whereas the glycosylation site in Ig1 of AXL is on β-strand A, on the opposite face of the Ig domain. Whether N-linked glycosylation contributes to ligand selectivity of TAM receptors remains to be established.

### The MERTK ECR has an extended, curved conformation in solution

We next assessed the solution conformation of the MERTK ECR using SAXS to address whether the conformation observed in the X-ray crystal structure is representative of what is seen in solution and presumably at the cell surface. We collected SAXS data on static samples using a home source system with concentrations ranging from 1.3 to 10 μM and SEC-SAXS at the BioCAT beamline (Advanced Photo Source, Argonne National Laboratory). Data collection and analysis details are in Supplementary Table S2. All data used in analysis showed linear Guinier regions (Figure 4A inset and Supplementary Figure S3D). The scattering at zero angle, *I*(0), indicates a monomeric protein, which is consistent with evaluation of the molecular weight using sedimentation equilibrium analytical ultracentrifugation (SE-AUC; Figure 5E and Table 1) and size-exclusion chromatography coupled to multiangle laser light scattering (SEC-MALLS; Supplementary Figure S3C).

**Figure 4:**
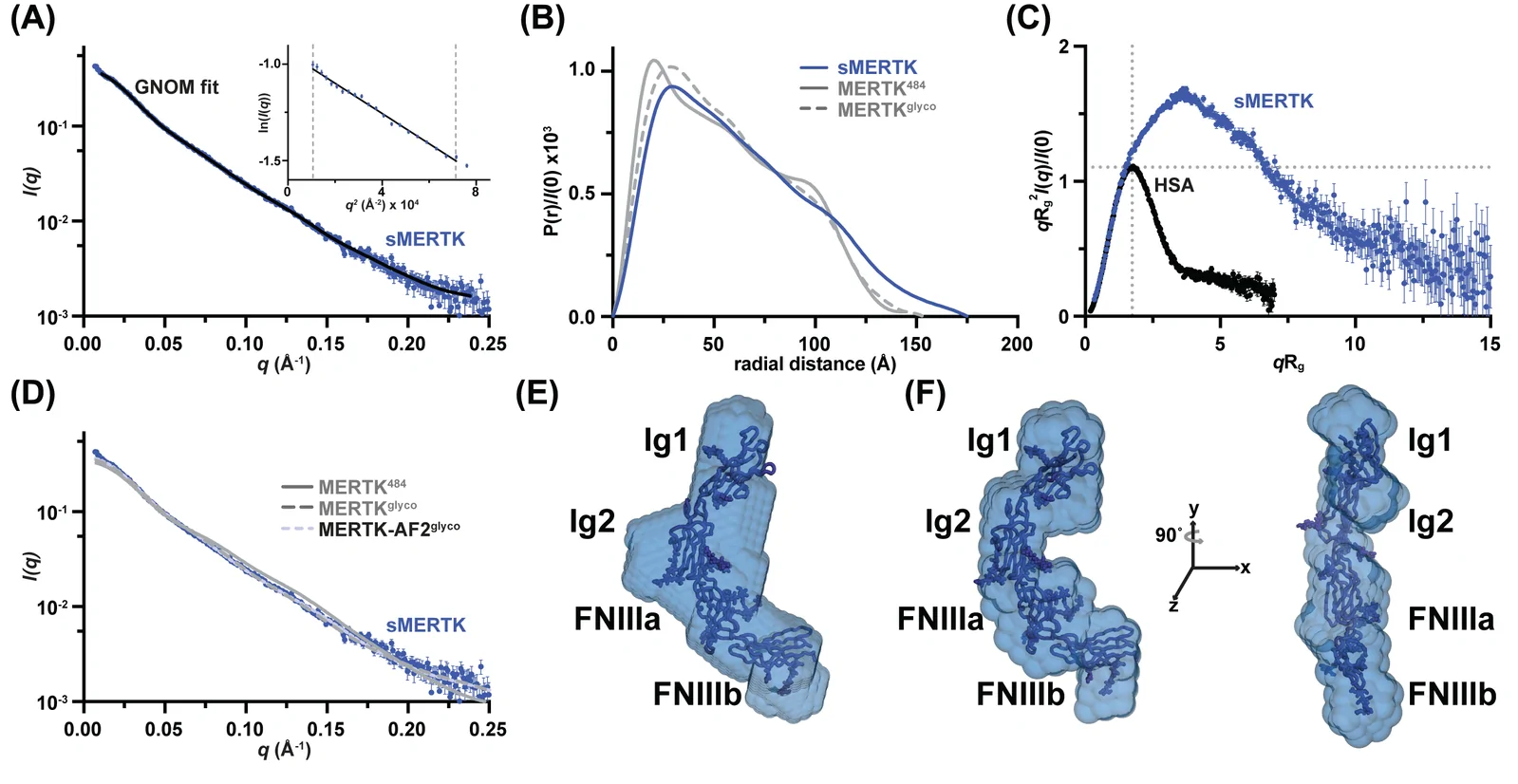
The solution conformation of the MERTK ECR (**A**) The SAXS scattering profile of sMERTK at 3.4 mg/ml is shown as intensity [*I*(*q*)] plotted against the momentum transfer *q* (*q* = 4πsinθ/λ, where 2θ is the scattering angle). Data points are indicated as blue circles. The black curve shows the GNOM [60] fit of the *P*(r) shown in panel (B). The Guinier plot for data where q*R_g_ < 1.3 is shown as an inset (upper right) plotted as the natural log of intensity against *q*^2^. Vertical dashed lines indicate the Guinier region. Residuals for the fit are shown in Supplementary Figure S3D. (**B**) The pair-distance distribution (*P*(r)) curve calculated from the scattering curves in panel (A) is shown as a blue line. The *P*(r) curves derived using CRYSOL [61] are shown for the X-ray crystal structure (MERTK^484^, solid gray line) and for a glycosylated model based on MERTK^484^ with Man_5_GlcNAC_2_ at 12 of the predicted N-linked glycosylation sites (MERTK^glyco^, dashed gray line). The maximal radial distance (*D*_max_) for sMERTK is significantly larger than for either of these models. See Table 1 and Supplementary Table S2 for SAXS structural parameters. (**C**) Dimensionless Kratky plot of data for sMERTK (blue circles) and for human serum albumin (HSA) control (black circles). Dotted lines are drawn at (*qR*_g_)^2^*I*(*q*)/*I*(0) = 1.104 and *qR*_g_ = 1.732 (3^1/2^). Compact globular proteins show a peak at the intersection of these lines. The broader, right-shifted peak for sMERTK is indicative of structural flexibility. (**D**) The same experimental scattering profile as plotted in panel (A) is shown in blue here compared with the theoretical scattering profiles for MERTK^484^, MERTK^glyco^, and the AF2 MERTK model to which Man_5_GlcNAC_2_ chains have been added to the same 12 N-linked glycosylation sites (MERTK-AF2^glyco^, light blue dashed line). See also Supplementary Figure S5B,C. (**E**) A low-resolution molecular envelope of the most probable model based on the scattering data shown in panel (A), calculated with the program DAMMIF [62], is shown in blue, superimposed with the MERTK^484^ structure, shown in ribbon representation with glycans in sticks. (**F**) Two orthogonal views of the refined molecular envelope generated following analysis of multiple DAMMIF results using DAMAVER [63] and DAMMIN [64] superimposed with the same MERTK^484^ model shown in panel (E). See the ‘Methods’ section and Supplementary Table S2 for details on bead model reconstructions.

**Figure 5:**
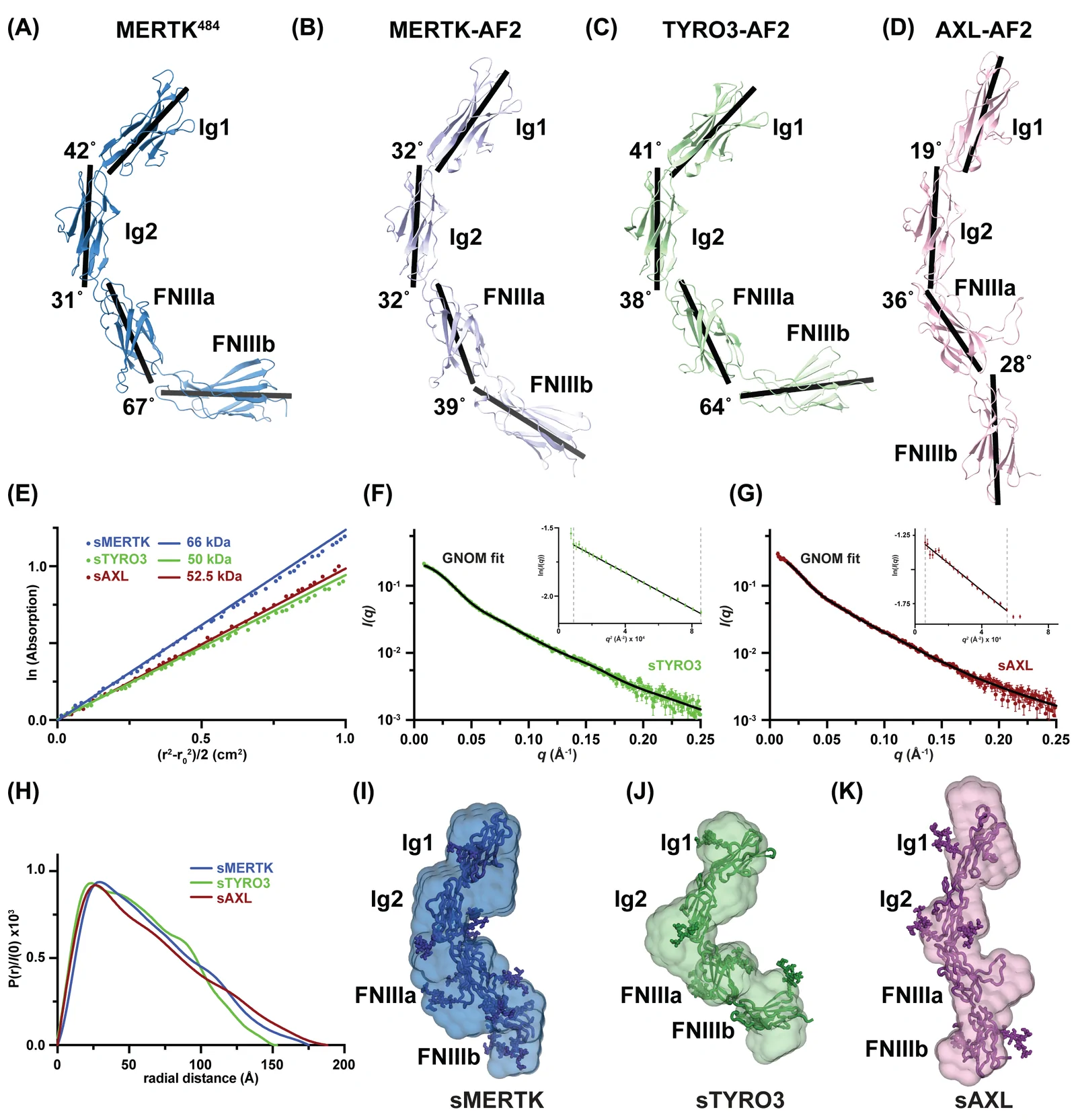
Each TAM ECR has a distinct curvature (A–D) A cartoon representation of MERTK^484^ (blue) is shown alongside similar representations of the AF2 models of each TAM receptor ECR, aligned based on the coordinates of Ig2 only. In each case the domains are labeled and interdomain angles indicated. Structures shown are (**A**) MERTK^484^ in blue, (**B**) MERTK-AF2 in pale blue, (**C**) TYRO3-AF2 in pale green, and (**D**) AXL-AF2 in pale pink. Axis (black rods) and interdomain angles were calculated using ChimeraX [65]. (**E**) Representative SE-AUC data at 12,000 rpm (circular data points) for sMERTK (blue) at 8 μM, sTYRO3 (green) at 13 μM and sAXL (dark red) 9 μM plotted as the natural logarithm of absorbance against radius, plotted as (*r*^2^ − *r*_0_^2^)/2 where *r*_0_ is the radial position of the meniscus. The lines indicate theoretical slopes for the indicated molecular weights, corresponding to those predicted for each monomeric sTAM. SAXS scattering profiles for (**F**) sTYRO3 and (**G**) sAXL, represented exactly as in Figure 4A. (**H**) The pair-distance distributions (*P*(r)) curve calculated from the scattering curves for sMERTK (as in Figure 4B), sTYRO3 and sAXL. (I–K) Refined molecular envelopes generated following analysis of multiple DAMMIF results using the programs DAMAVER [63] and DAMMIN [64], as described in the ‘Methods’ section. (**I**) The same molecular envelope as shown in Figure 4E but here superimposed with the MERTK-AF^glyco^ model. (**J**) The refined molecular envelope from analysis of sTYRO3 scattering data is shown in green superimposed on TYRO3-AF^glyco^ model. (**K**) The refined molecule envelope from analysis of sAXL scattering data is shown in green superimposed on AXL-AF^glyco^. See the ‘Methods’ section and Supplementary Table S2 for details on bead model reconstructions.

**Table 1:** Size and shape of sTAMs

Sample:	sMERTK	sAXL	sTYRO3
Molecular weights (kDa)^1^			
Estimated from sequence (including glycans^2^)	65.8	52.4	49.6
SAXS *I*(0) compared with standard	68	58 ± 4	50 ± 5
SEC-MALLS	64 ± 1	53 ± 1	51 ± 3
SE-AUC	65 ± 1	56 ± 3	49 ± 3
SAXS structural parameters^3^			
Guinier *R*_g_ (Å)	49 ± 1	52 ± 3	44 ± 1
GNOM [60] *R*_g_ (Å)	51.4 ± 0.6	55 ± 2	47.5 ± 0.7
*D*_max_ (Å)	176 ± 1	187 ± 1	153 ± 1

1 Experimental molecular weight determinations are the mean and standard deviation from independent biological replicates (see the ‘Methods’ section for details).

2 Including Man_5_GlcNAc_2_ (mwt 1216 Da) at 12, 5, and 6 glycosylation sites for sMERTK, sAXL, and sTYRO3, respectively.

3 Data here are mean and standard deviation over independent biological replicates (see the ‘Methods’ section for details). For errors of fitting for representative datasets, see Supplemental Table S2. Similar parameters have previously been reported for mouse and human sAXL [66].

Analysis of the SAXS data shows clear indication of an extended and flexible molecule (Figure 4 and Table 1). The pair-distance distribution (*P*(r)), calculated from the indirect Fourier transform of the scattering data, gives a weighted distribution of interatomic lengths in the molecule. For sMERTK, experimental *P*(r) curves (Figure 4B, blue curve) indicate a maximal dimension (*D*_max_) of 175.5 ± 0.7 Å and radius of gyration (*R*_g_) of 51.4 ± 0.6 Å (Table 1 and Supplementary Table S2). These values are substantially larger than those calculated from the X-ray crystal structure (designated MERTK^484^) using the program CRYSOL [61]—152 and 45.3 Å for *D*_max_ and *R*_g_, respectively, which yields a poor match to the experimental scattering data (Figure 4B,D and Supplementary Table S2).

One confounding issue in evaluating the size and shape of sMERTK is its high degree of N-linked glycosylation, as described above. To generate a fully glycosylated sMERTK model (MERTK^glyco^), we added or extended the glycans, using the program GLYCAM [67], so that there were chains of two N-acetylglucosamine (GlcNAc) and five mannose (Man) units at twelve of the predicted N-linked glycosylation sites. This chain length was selected based on experimentally derived molecular weights for sMERTK (Table 1) that suggest 15%–25% glycan by mass. The calculated *P*(r) curve for MERTK^glyco^ more closely resembles that of the experimental sMERTK data, with a χ^2^ value for the fit to the experimental data of 1.1–1.4 (Figure 4B,D). The *D*_max_ and *R*_g_ remain smaller than we observe experimentally (Table 1 and Supplementary Table S2), which could be due, at least in part, to the N-terminal ∼70 disordered amino acids.

The Kratky plot (Figure 4C) has a significantly right-shifted (to a higher *qR*_g_) peak compared with a globular standard (HSA), although the curve tends toward zero as *qR*_g_ increases. This is consistent with a multi-domain protein that has flexibility between each globular domain [68]. We asked whether approaches that account for flexibility and/or disorder to model SAXS data, such as the program SREFLEX [69], would improve fits to our experimental data. The χ^2^ values for the fits were modestly reduced using these approaches, and the models were consistent with interdomain flexibility, particularly of Ig1 and FNIIIb relative to an Ig2/FNIIIa core. Taken together, these data suggest that sMERTK has some flexibility and explores more extended conformations in solution than we observe in the crystal structure.

Flexibility of MERTK is further supported by a relatively high ambiguity score of ∼2.6 from the program AMBIMITER [70], which indicates that there are multiple conformations consistent with the scattering profile. We nevertheless evaluated whether information on the sMERTK conformation may be gleaned by using the program DAMMIF [62] to calculate bead models that are consistent with the scattering data. On average for sets of 40 calculations, each bead model fit the data well with χ^2^ values close to one. The normalized spatial discrepancy across models was 0.85, suggesting that all models are relatively similar. The most probable model (Figure 4E) fits the experimental data with a χ^2^ value of 1.1 and has an normalized spatial discrepancy (NSD) value of 0.78. Refinement of the surface of the models to optimize the fit to the data results in an envelope that is an improved match with *D*_max_ (174.3 Å) and *R*_g_ (51.3 Å), close to the experimental values (Figure 4F and Table 1), and a shape that is consistent with the curvature of the X-ray crystal structure.

### Each TAM receptor has a unique curvature in its ECR

MERTK is one member of a family of three closely related RTKs that comprise the TAM receptor family, the other members being TYRO3 and AXL. AF2 [35,71] predictions suggest all three TAM ECRs form extended, curved structures, with each receptor having a distinct curvature—TYRO3 is the most bent, MERTK intermediate, and AXL relatively straight (Figure 5B–D and Supplementary Figure S4). The Ig2/FNIIIa core shows high pLDDT scores in each case, with scores predominantly >90, other than a few surface loops (mostly in FNIIIa). The pairwise main chain RMSDs for all Ig2 structures (X-ray crystal structures and AlphaFold predictions) are ∼0.6 Å, and we use this domain to overlay all models. Overlaid in this way, the variability in the relative Ig1 and FNIIIb orientations in an ensemble of five AF2 predictions is apparent (Supplementary Figure S4E–G), although in all cases the trend is always that AXL is relatively straight and TYRO3 most curved. N-linked glycans are not modeled in these AF2 calculations, although it is of note that several of the predicted sites of N-linked glycosylation in the AF2 models for TYRO3 and AXL fall close to domain boundaries. For example, N43 of AXL at the Ig1/Ig2 interface and N293 of TYRO3 at the FNIIIa/FNIIIb boundary (Supplementary Figure S4C,D), which we predict could stabilize the orientations of Ig1 and FNIIIb, respectively, relative to the Ig2/FNIIIa core, as we see for the glycans on N170 and N294 for MERTK^484^.

The AF2 MERTK (MERTK-AF2) model is overall less bent than our X-ray crystal structure, with the largest difference being the position of FNIIIb with respect to FNIIIa. The interdomain FNIIIa-FNIIIb angle is 39° in the AF2 model (AF-Q12866-F1-v6) compared with 67° in our structure. This may be due to crystal packing contacts between FNIIIb and the Ig2 of a symmetry molecule (Figure 2C and Supplementary Figure S2C,D), but it could also be a favored conformation for the glycosylated MERTK. The different curvature of the MERTK-AF2 does not lead to a better fit to the experimental SAXS data. As for the crystal structure, addition of glycan chains improves the fit (Figure 4D, Supplementary Figure 5B, and Supplementary Table S2). The uncertainty in contribution from the glycan chains on FNIIIb (two modeled in MERTK-AF^glyco^) makes it difficult to discriminate between these two relatively similarly bent MERTK conformations based on our SAXS data.

To address whether TYRO3 is indeed more bent and AXL straighter in solution, we collected SAXS data for secreted versions of these two TAM receptors and compared these data to our observations with MERTK. We first established that the TYRO3 and AXL ECRs are monomeric in solution, as we observed for MERTK. SE-AUC and SEC-MALLS data for each of the sTAM receptors indicated monomeric proteins (Figure 5E, Supplementary Figure S3C, and Table 1). Consistent with this, the SAXS scattering at zero angle, *I*(0), shows no indication of any oligomerization or aggregation (Figure 5F,G, Supplementary Figure S3E,F, and Table 1).

There are clear differences in the *P*(r) curves generated from experimental SAXS scattering profiles for sMERTK, sTYRO3, and sAXL (Figure 5H), with sAXL showing the largest *D*_max_ (184 ± 3.5 Å) and sTYRO3 the most compact (*D*_max_ 151 ± 2 Å; Table 1 and Supplementary Table S2). As for sMERTK, the AlphaFold models are poor fits to the experimental scattering data, but fits are substantially improved when glycan chains are modeled on the predicted glycosylation sites, exactly as we did for MERTK (see the ‘Methods’ section for details). Metrics of the sAXL and sTYRO3 scattering data suggest flexible structures. The peaks in the Kratky plot for sTYRO3 and sAXL are at higher *q*R_g_ than for sMERTK, suggesting a greater degree of flexibility for these two receptor ECRs (Supplementary Figure S3G), perhaps reflecting the lower degree of glycosylation of TYRO3 and AXL compared with MERTK (Figure 3A). We nevertheless asked whether we could discern the differences in curvature predicted for each TAM ECR from our SAXS data. Using exactly the same procedure as for MERTK, we generated bead models based on the sTYRO3 and sAXL data. As shown in Figure 5I–K, the refined bead models show a remarkable consistency with the curvature predicted from AF2 models, with sTYRO3 showing more curvature than for sMERTK and sAXL being relatively straight (details provided in Supplementary Table S2).

### Implications for TAM receptor activation

The data we present here provide experimental evidence that the ECRs of TAM receptors are extended with flexibility that is limited by glycans at domain interfaces. MERTK and TYRO3 are significantly bent, whereas the long axis for AXL is relatively straight (Figure 5). This grouping of the three TAM receptors, with MERTK and TYRO3 more similar and AXL distinct, correlates, at least in part, with differences in activation and signaling for these receptors [13,14,31,72,73]. At the level of ligand binding, this grouping is clear. GAS6 binds to AXL with nanomolar affinity, whereas it binds to MERTK and TYRO3 approximately 250- and 50-fold more weakly, respectively [32]. We confirmed these binding trends with our soluble sTAMs using surface plasmon resonance (SPR; Figure 6A,B).

**Figure 6:**
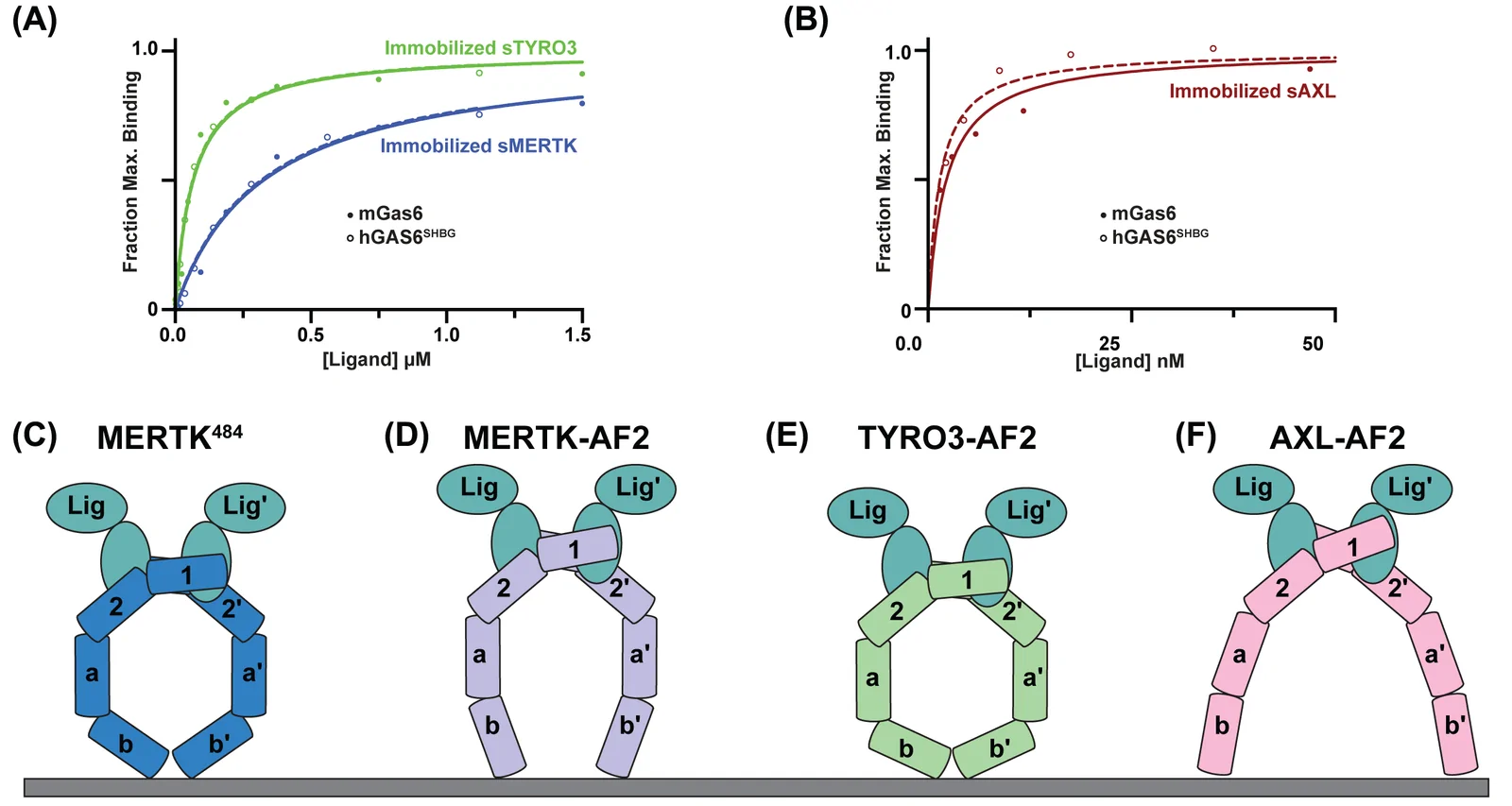
Possible models of ligand-induced TAM receptor dimers (**A**) SPR analysis of the binding of mouse Gas6 (mGas6) and hGAS6^SHBG^ to immobilized sMERTK (blue) and sTYRO3 (green). The equilibrium SPR responses for samples of the indicated concentrations of ligand are normalized to the maximal SPR response. The curve shows the fit of these normalized data to a simple one-site Langmuir binding equation. As expected, there is no difference in binding for the isolated SHBG compared with the full-length mGas6, and sTYRO3 binds approximately five-fold more tightly than sMERTK. A similar trend was seen for the binding of sTAMs to immobilized mGas6, hGAS6, and hGAS6^SHBG^. (**B**) SPR analysis of the binding of mGas6 and hGAS6^SHBG^ to immobilized sAXL (dark red) presented as in A. These data confirm that this ligand binds to sAXL with low nanomolar affinity. Similar results were obtained using immobilized mGas6, hGAS6, and hGAS6^SHBG^. (C–F) Cartoons representing hypothetical TAM receptor dimers based on the GAS6/AXL crystal structure (PDB: 2C5D) [45] (Figure 2B) indicate a potential impact of the different curvatures of each TAM ECR. One copy of the relevant TAM ECR model was overlaid on each of the two AXL protomers in the PDB: 2C5D using the coordinates of only Ig2. No additional alterations in domain positions are introduced in these hypothetical models but would be expected to accommodate differences in ligand binding for each TAM. The gray bar indicates the approximate location of the plasma membrane. In each case the ligand is represented by two teal ovals (Lig and Lig′), and the receptor domains are colored as in Figure 5A–D. Domains are labeled (1/1′ for Ig1, 2/2′ for Ig2, a/a′ for FNIIIa, and b/b′ for FNIIIb). (**C**) The model based on MERTK^484^ places the C-terminal ends of the two FNIIIb domains ∼50 Å apart. For the AlphaFold models, these distances are (**D**) ∼90 Å MERTK-AF2, (**E**) ∼25 Å for TYRO3-AF2, and (**F**) ∼120 Å for AXL-AF2.

We wondered whether the ECR conformation may play a role in directing ligand-induced differences. The precise mechanism of activation of TAM receptors remains unknown, but it is assumed that ligand-induced receptor dimerization plays a role, as it does for most RTKs [74]. This is strongly supported by the X-ray crystal structure of the first two Ig domains of AXL bound to the two LG domains of GAS6 [45], which shows a ligand-mediated dimer (Figure 2B). We wondered what impact the curvature of the TAM ECRs has on models of ligand-bound complexes based on this GAS6/AXL dimer. Using coordinates of Ig2 only, we superimposed onto the GAS6/AXL structure our crystal structure of MERTK and the AF2 models of each TAM receptor. Cartoons for the resulting hypothetical ligand-bound complexes are shown in Figure 6C–F. For MERTK and TYRO3, the curvature of the two ECRs brings the C-terminal ends toward each other so the ends of the two FNIIIb domains are approximately 25 and 50–90 Å apart. By contrast, the straighter AXL ECR places these two domains ∼120 Å apart. If these separations constrain the positions of the intracellular kinase domains, it would be predicted that simple ligand-induced dimerization would have minimal effect on the transactivation of the intracellular kinase domains for AXL but may have a more significant effect for TYRO3 and, perhaps, MERTK. It is estimated that most GAS6 is pre-bound to AXL in cells and tissues [12,30,75] yet, in most settings, AXL remains inactive without an additional stimulus from a PtdSer-containing membrane, vesicle, or virus. There appears to be a distinction between AXL and the other two TAM receptors in the requirements and responses to exposure to different PtdSer levels, although there is no clear consensus on the mechanistic details [14–16,31]. Differences at the level of ECR conformations may provide a framework for regulation of distinct TAM receptor signaling.

## Discussion

We and others have previously shown that the membrane-proximal FNIII domains of TIE2 mediate ligand-independent dimerization [20,21]. Moreover, ligand-independent dimers of TYRO3 have been reported [43]. Our initial studies of the TAM receptor ECRs were therefore motivated, at least in part, by the possibility that TAM receptors might similarly form ligand-independent dimers. However, in SE-AUC, SEC-MALLS-, and SAXS analysis of the TAM ECRs, we find no evidence for homodimerization in the absence of ligand up to concentrations exceeding 100 μM (Table 1, Figure 5E, and Supplementary Figure S3C–F). GAS6 induces only very weak dimerization of the AXL ECR (Supplementary Figure S3H,I), as has previously been reported [45].

The MERTK crystal structure nevertheless suggests a possible homomeric interaction (Figure 2 and Supplementary Figure S2C,D) with interesting parallels to interactions observed in a third RTK family with membrane-proximal FNIII domains—the Eph receptors [19]. Higher-order receptor assembly is an important component of Eph receptor activation and regulation [19]. In several family members, including EphA2, EphB2, EphA4, and EphB6, head-to-tail interactions have been described in which the ligand-binding region of one receptor interacts with the membrane-proximal FNIII domain of another molecule [23,36,37,76]. Such interactions can extend receptor assemblies beyond the ligand-bound dimer and have been proposed either to promote or constrain signaling, depending on the receptor and context [23,36,37]. More broadly, studies of receptor tyrosine pseudokinases, including EphB6, illustrate how receptor–receptor interactions and higher-order organization can provide non-catalytic regulatory functions [77].

Formation of higher-order assemblies or clusters may similarly be a component in the activation mechanism of TAM receptors, with receptor organization promoted by engagement of membrane PtdSer via the N-terminal Gla domain of the TAM receptor ligands [12]. A PtdSer-dependent clustering mechanism has been proposed for AXL [16], in which the bipartite binding of GAS6 to AXL (Figure 2B) through sites of differing affinity provides a system where local ligand concentration can drive receptor activation [45]. In this context, the absence of detectable MERTK ECR dimerization in solution does not necessarily exclude a role for the interaction observed in the crystal structure. A weak receptor–receptor interaction could become significant when receptors are confined to the membrane and brought into proximity by ligand, PtdSer engagement, or other mechanisms of receptor clustering. We therefore speculate that the head-to-tail interaction observed in the MERTK crystal structure, between Ig2 of one receptor and the membrane-proximal FNIII domain of another, could contribute to the organization or regulation of MERTK assemblies. The distinct ECR curvatures observed for the three TAM receptors could further influence the accessibility and geometry of such interactions, potentially providing receptor-specific constraints on higher-order assembly. Whether related interactions occur in AXL or TYRO3 and whether they promote or constrain receptor activation will require further investigation.

## Methods

### Reagents and ligands

Human GAS6 (hGAS6) was purchased from R&D Systems (Cat. #885-GSB), and human protein S was purchased from Prolytix (Cat. #HCPS-0090). HSA for use as SAXS standard was purchased from Sigma−Aldrich (Cat. #A8763), and bovine serum albumin (BSA) for SEC-MALLS was from Pierce (Thermo Scientific Cat. #23209).

### Protein expression and purification

The coding regions for the ECRs of MERTK, AXL, and TYRO3 were inserted into pFastbac1. For each receptor, each pFastbac1 plasmid encodes the native signal peptide and a C-terminal His_6_ tag that follows amino acid 484 for MERTK, 447 for AXL, and 427 for TYRO3 (ending 18, 3, and 2 amino acids, respectively, before the start of the transmembrane (TM) domain, predicted using the DeepTMHMM server [78]). The C-terminal truncation at 484 for MERTK was optimal for crystal growth. A longer version of MERTK that ends after amino acid 505 (3 amino acids from the TM domain) was also generated and behaved similarly to the C-terminally truncated version. All experiments reported in this manuscript use the shorter protein. Insect cell expression of these soluble TAM ECRs (sTAMs) was induced by infection with appropriate recombinant baculovirus of 4–7 l of *Spodoptera frugiperda* Sf9 cells at a density of ∼2.5 × 10^6^ cells/ml. Sf9 cells were propagated at 27°C with constant shaking at 110–120 rpm in ESF 921 serum-free insect cell culture medium (Expression Systems) with or without 50 U/ml penicillin/streptomycin. Two to three days post-infection, clarified conditioned media was concentrated ∼2-fold, diafiltered against 3.5 volumes of 25 mM Tris–HCl, 150 mM NaCl, pH 8.0 (buffer A) using a 30 kDa cutoff Prep/Scale-TFF (MilliporeSigma), cartridge and further concentrated to <1000 ml for loading onto a 2–10 ml Ni-Penta agarose column (Marvelgent Biosciences). Following extensive washing with buffer A plus 10 mM imidazole, the protein was eluted by sequentially washing with two column volumes of buffer A containing 25, 50, 75, 150, and 300 mM imidazole, pH 8.0. Fractions containing the desired sTAM were diluted 4–6-fold with 25 mM Tris–HCl, pH 8, and loaded on a 5 ml Source 15Q (Cytiva) ion exchange column. Protein was eluted with a gradient of NaCl (25–500 mM over 40 column volumes) and protein-containing fractions concentrated and further purified on a Superose 6 size exclusion column (Cytiva) equilibrated in 25 mM HEPES, 150 mM NaCl, pH 8.0, or 25 mM Tris–HCl, 25 mM NaCl, pH 8.0.

Recombinant full-length mGas6 was generated in HEK293-EBNA cells exactly as described [14]. The conditioned medium was applied directly to a Ni-Penta agarose column, and the protein was subsequently purified exactly as for sTAMs. A fragment of human GAS6 containing just the SHBG domain (amino acids 261–678; hGAS6^SHBG^), with the native signal peptide and a C-terminal His_6_ tag, was expressed and purified exactly as for sTAMs.

### X-ray crystallography

#### Crystallization

The sMERTK in 25 mM Tris, 25 mM NaCl, pH 8.0, was concentrated to 4–15 mg/ml and crystallized using the hanging drop vapor diffusion method at 20°C. Preliminary crystals were obtained from multiple conditions in sparse and grid screens (including Hampton Research Crystal Screen, Index, and PEG/Ion) using 200 nl drops (1:1 mixture of protein:reservoir) incubated over 100 μl of reservoir. Following optimization, single crystals were obtained in 1 μl drops (1:1 mixture of protein:reservoir) using a reservoir of 20% (w/v) PEG 3350, 8% Tacsimate, pH 8.0. Crystals were flash-frozen directly from the drop. Crystals belonged to space group P2_1_2_1_2_1_ and diffracted to 3.4 Å resolution.

#### Data collection and structure determination

Data were collected at APS beamline 23-ID-D using a PILATUS 6M detector. Data were reduced with XDS [79,80] and scaled using the CCP4 package [81]. Data collection statistics are summarized in Supplementary Table S1. The structure was solved by the method of MR using the program PHASER [82] implemented in Phenix [83]. Initial maps were obtained using individual Ig and FNIII models created with the Phyre2 server [38] based on PDB: 1RHF [43] and PDB: 2DBJ [47], respectively, plus a trimmed polyalanine Ig domain model based on PDB: 2C5D [45]. Subsequently, MR was repeated with an AF2 [35,71] model. Searching with the intact AF2 ECR failed. The best solution using the AF2 models was obtained by searching for the tandem Ig2-FNIIIa fragment, plus the individual Ig1 and FNIIIb domains. The structure was refined by cycles of model building in COOT [84,85] and refinement in Phenix [83], BUSTER [86], and the PDB-REDO webserver [87]. Glycans were added using the carbohydrate module in COOT [88]. Multiple sugar units could be modeled at positions N114, N170, N207, N215, N234, N294, N316, N354, and N395 (Figure 3 and Supplementary Figure S2A,B). Only one NAG could be modeled at N389, and for N329 and N336, although density could be seen, we were not able to reliably place a NAG group. No density could be seen for any sugar groups at N442 or N454, although this does not exclude the possibility that these sites are glycosylated. Structure figures were made using PyMOL [89] or ChimeraX [65].

### Small-angle X-ray scattering

SAXS data collected at Yale were recorded at 4°C on a Rigaku BioSAXS-2000 nano 2D Kratky block camera system with a Rigaku 007HF rotating anode source and a Rigaku HyPiX-3000 HPAD CCD detector, with 90-min exposures and frames recorded every 10 min. Data were collected using SAXLab version 4.0.2 (Rigaku). Protein concentrations ranged from 2.5 to 12 mg/ml in 10 mM HEPES, 150 mM NaCl, pH 8.0, or 25 mM Tris, 25 mM NaCl, pH 8.0. Samples, along with matched buffer controls and molecular weight standards (HSA) at 1.25–4.75 mg/ml), were loaded into a quartz capillary using a Rigaku automatic sample changer with an extensive wash cycle between samples. SEC-SAXS for sMERTK and sAXL was performed at BioCAT beamline 18ID at the Advanced Photon Source, Argonne National Laboratory, equipped with a custom HPLC system (Agilent). Samples were loaded on a Superdex 200 10/300 Increase column (Cytiva) equilibrated in 25 mM Tris, 25 mM NaCl, pH 8.0 using a flow rate of 0.6 ml/min with in-line MALLS (see below) and a quartz capillary SAXS flow cell (1.0 mm inner diameter, 20 μm thick walls). Scattering data were collected using a Pilatus3 X 1M detector (Dectris) placed 3.651 m from the sample. Exposures of 0.5 s were acquired every 1 s during elution. Buffer blanks were created by averaging regions flanking the elution peak and subtracted from exposures selected from the elution peak. SAXS data collection parameters are summarized in Supplementary Table S2.

In all cases data reduction and buffer subtraction were performed with BioXTAS RAW [90] to yield final scattering profiles in which intensity, *I*, is plotted as a function of *q* (4πsin*θ*/λ, where 2*θ* is the scattering angle). All samples were monodisperse, as evidenced by linear Guinier regions (Supplementary Figures S3D–F and S5J,K). Values for the scattering intensity extrapolated to zero angle, *I*(0), calculated from the Guinier region where q*Rg <1.4, varied linearly with protein concentration. Molar mass was determined relative to HSA using data collected on the same day or within one month of relevant samples. The distance distribution function *P*(r) was calculated using the Fourier inversion of the scattering intensity *I*(*q*) using GNOM [60] implemented in BioXTAS RAW [90]. The likely ambiguity in a shape reconstruction based on the scattering data was estimated using the program AMBIMETER [70]. Structural parameters are summarized in Supplementary Table S2, and the mean and standard deviation across biological repeats are listed in Table 1 (*n* = 3 for sTYRO3 and sAXL, *n* = 2 for sMERTK (except *I*(0), which was determined only once for sMERTK)).

*Ab initio* bead density shape envelope models were generated using the ATSAS suite of programs [91] implemented in the RAW package [90] or using ATSAS online. Forty independently calculated models were generated using the program DAMMIF [62] without symmetry constraints or a starting model, and the models were analyzed with DAMAVER [63]. Envelopes were consistently generated with good correlations to experimental scattering data (χ^2^ values 0.91–1.29). NSD values were relatively high, ranging from 0.85 ± 0.008 to 1.285 ± 0.002. Consistent with this, the models were filtered into 3–11 related structures. Shape model-fitting results are summarized in Supplementary Table S2.

### Additional analysis of molecular weight

#### Sedimentation equilibrium analytical ultracentrifugation

SE-AUC experiments were performed on a Beckman XL-I analytical ultracentrifuge (Beckman, Fullerton, CA) at 20°C using an An Ti 60 Ti rotor at speeds ranging from 5000 to 12,000 rpm, with at least three different speeds per experiment. Samples at three different concentrations (ranging from 2 to 10 μM) were loaded, along with buffer-matched controls, in 6-channel charcoal-Epon cells with quartz windows and a path length of 1.2 cm. Following sample sedimentation to equilibrium (∼17 h) at each speed, radial absorption data were collected at 280, 285, 290, or 295 nm (depending on the experiment). To establish that equilibrium had been reached at each speed, successive scans were taken with 2 h separation to confirm that no further difference was seen. The resulting data (three speeds and three concentrations) were fit globally to determine the ideal molecular weight using the program HeteroAnalysis (v1.1.60, written by J. Cole and J. Lary, University of Connecticut), and these experimental molecular weights were compared with the expected values to assess the formation of any oligomeric species. The partial specific volume of sTAMs was estimated as 0.71 ml/g. The natural logarithm of the absorbance is plotted as a function of the radial position expressed as (*r*^2^ − *r*_0_^2^)/2, where *r*_0_ is the position of the meniscus. For a homogeneous sample, this representation yields a straight line with a slope proportional to the molecular weight. The mean and standard deviation of the fitted molecular weight across three biological repeats are listed in Table 1.

#### Size exclusion chromatography coupled to multiangle laser light scattering

SEC-MALLS and differential refractive index detection was performed at Yale using an Agilent 1260 Infinity Pump with a Zenix SEC 300 guard column (3 μm, 4.6 × 20 mm) and ZeniX-C-SEC 300 column (3 μm, 4.6 × 300 mm) (Sepax) equilibrated with 10 mM HEPES, 150 mM NaCl, pH 8.0 or 25 mM Tris, 25 mM NaCl, pH 8.0 at a flow rate of 0.35 ml/min at 20°C, and coupled with a DAWN HELEOS II detector (Wyatt Technology) using a laser emitting at 661 nm and an Optilab T-rEX detector (Wyatt Technology). A 10 μl sample of protein at ∼30 μM was injected for analysis. SEC-MALLS data were also collected at the BioCAT beamline 18ID using a custom Agilent HPLC with a Superdex 200 10/300 Increase column (Cytiva) equilibrated in 25 mM Tris, 25 mM NaCl, pH 8.0 using a flow rate of 0.6 ml/min and the same model of Wyatt detectors. Data were analyzed using ASTRA 7.1.3 software (Wyatt Technology). Samples of 300 μl of sMERTK (5 mg/ml) and sAXL (3.6 mg/ml) were loaded on to the SEC column. A sample of sTYRO3 at 1 mg/ml yielded an unstable baseline and is not shown in Supplementary Figure S3C (SAXS data were also not recorded for this sample). Normalization was done using BSA. The refractive-index increment for each sample was estimated based on the mass fraction of glycans and assuming a refractive-index increment (dn/dc) of 0.185 and 0.145 ml/g for protein and glycans, respectively. Resulting dn/dc values were 0.176 ml/g for sMERTK, 0.179 ml/g for sTYRO3, 0.180 ml/g for sAXL, 0.184 ml/g for recombinant mGas6, and 0.183 ml/g for a 1:1 complex of this Gas6 with sAXL. The mean and standard deviation (*n* = 2) for the weight-averaged molar weights of sTAMs are summarized in Table 1.

### Surface plasmon resonance

SPR experiments were carried out on either a Biacore 3000 or T200 instrument at 25°C using a running buffer of 10 mM HEPES, 150 mM NaCl, 3 mM EDTA, 0.005% Tween-20, pH 8.0 (buffer B). CM5 chips were activated using the Cytiva amine coupling kit. For ligand immobilization, hGAS6, mGas6, and hGAS6^SHBG^ at 4 μg/ml in 10 mM sodium acetate pH 5.0 were flown over activated CM5 surfaces at 5 μl/min for 10 min to achieve 600–2000 response units of immobilized ligand. A series of sTAM samples (in buffer B) of different concentrations (up to 500 nM for sAXL and up to 5 μM for sMERTK and sTYRO3) were passed over these immobilized ligands at 5 μl/min for 50 min for sAXL and 10 min for sMERTK and sTYRO3. Alternatively, sTAMs were amine coupled to CM5 chips using 4 μg/ml sMERTK in 10 mM sodium acetate, pH 6.0, and 20 μg/ml and 5 μg/ml for sTYRO3 and sAXL, respectively, in 10 mM sodium acetate pH 5.0. Samples of mGas6 and hGAS6^SHBG^ were flown over these surfaces at concentrations up to 1.2 μM at a flow rate of 5 μl/min for 50 min. For protein S, samples up to 4.6 μM were applied for 10 min at 5 μl/min. Regeneration to baseline values following each injection was achieved either by time or using two 10 μl injections of 2 M NaCl in 10 mM sodium acetate pH 5.0. Signals were corrected by subtraction of the SPR signal from a control surface that was activated and then blocked with ethanolamine. The saturation equilibrium SPR response for each sample was converted to the fraction of maximal binding, plotted against the analyte concentration, and these curves were fit to a simple one-site binding model using Prism 9 (GraphPad Software, Inc.). Plots show data normalized to the maximal saturation binding response. Similar trends in binding affinity were seen for all experiments.

### Structure analysis

#### Generation of AlphaFold and glycosylated models

The AF2 models of MERTK (Uniprot ID: Q12866), TYRO3 (Uniprot: Q06418), and AXL (P30530) were downloaded from the AlphaFold Protein Structure Database with the identifiers AF-Q12866-F1-v6, AF-Q06418-F1-v6, and AF-P30530-F1-v6, respectively. The range of amino acids used in structural analysis, along with the confidence scores (pLDDT) is shown in Supplementary Figure S4B–D). Additional models were generated using ColabFold [92] to visualize the variability in predicted conformations. The regions from such models corresponding to the amino acids in the expressed sTAMs are shown in Supplementary Figure S4E,F. AlphaFold3 [93] models calculated using the full-length proteins (including the TM and intracellular region) or with just the ECR also show the same trend in the curvature of the three ECRs, with AXL the least bent, MERTK intermediate, and TYRO3 the most curved.

Sites of N-linked glycosylation were predicted using the NetNGlyc-1.0 webserver (https://services.healthtech.dtu.dk/services/NetNGlyc-1.0/) [48]. To generate glycosylated models, the program GLYCAM (Woods Group. (2005–2026) GLYCAM Web Glycoprotein Builder. Complex Carbohydrate Research Center, University of Georgia, Athens, GA (http://glycam.org)) was used to place glycan chains of MERTK^484^ and AF2 models for each TAM receptor. Based on our molecular weight analysis of sMERTK, we opted to add the high mannose Man_5_GlcNAc_2_ (α-D-Mannopyranosyl-(1->3)-[α-D-mannopyranosyl-(1->3)-[α-D-mannopyranosyl-(1->6)]-α-D-mannopyranosyl-(1->6)]-β-D-mannopyranosyl-(1->4)-2-acetamido-2-deoxy-β-D-glucopyranosyl-(1->4)-2-acetamido-2-deoxy-β-D-glucopyranose). Models were optimized using the GLYCAM06 force field [94]. For MERTK^484^, additional sugar groups were added to those modelled based on the experimental electron density maps (at N114, N170, N207, N215, N234, N294, N316, N354, N389, and N395), and glycan chains were added at N329 and N336. These same sites were decorated for the MERTK-AF2 model. For AXL-AF2, glycans were added at N43, N157, N198, N339, and N401, and for TYRO3-AF2, glycans were added at N63, N191, N230, N293, N366, and N380. Glycans chains were omitted at N442 and N454 in MERTK based on analysis of the experimental electron density maps. For AXL and TYRO3, we omitted glycans at N345 and N240, respectively, as the sites of lowest probability for glycosylation from NetNGlyc-1.0 [48]. In each case, this level of glycosylation yields a predicted molecular weight that closely matched our experimentally determined values (Table 1).

#### Analysis of structural models

Structural overlays were performed with PyMOL [89], and buried surface areas were calculated with PDBePISA [95]. Interdomain angles were determined using UCSF ChimeraX [65] using axis defined based on domain selection. Searches for structural homologues were performed using the DALI webserver [39] using both the PDB and AF-DB searches.

#### Comparison of structural models to SAXS scattering profiles

The ATSAS program CRYSOL [61] was used to generate theoretical scattering profiles from atomic models and to evaluate how well these profiles match the experimental data. For the AF2 models, the amino acid ranges used for this analysis were 93–484 for MERTK, 41–415 for TYRO3, and 33–427 for AXL. Coordinates were superimposed on experimental molecular envelopes using CIFSUP, part of the ATSAS package [91]. The ATSAS program SREFLEX [69] was used to assess whether the fit to the scattering data could be improved by allowing flexibility in domain arrangement. The contribution of the 72 amino acid N-terminal disordered region of sMERTK was addressed with SREFLEX and the ensemble optimization method [96], neither of which provided any additional information on MERTK conformation, presumably due, at least in part, to the uncertainty in the contribution of the glycans.

## Supplementary Material

Supplementary Figures S1-S5 and Tables S1-S2

## Data Availability

The atomic coordinates and structure factors for the MERTK crystal structure generated during the present study are available at the Protein Data Bank with accession code 37NT (pdb_000037nt) [97]. Any additional information required to reanalyze the data reported in the present paper is available from the lead contact upon request.

## Open Access

Open access for this article was enabled by the participation of Yale University in an all-inclusive Read & Publish agreement with Portland Press and the Biochemical Society.

## Abbreviations

AF2: AlphaFold2
BSA: bovine serum albumin
Eph: erythropoietin-producing human hepatocellular
ECR: extracellular region
FNIII: fibronectin type III
GAS6: human growth arrest-specific 6
Gla: γ-carboxyglutamate
GlcNAc: N-acetylglucosamine
HSA: human serum albumin
LG: Laminin G-like
mGas6: mouse growth arrest-specific 6
MR: molecular replacement
PtdSer: phosphatidylserine
RTK: receptor tyrosine kinase
RMSD: root mean square deviation
SE-AUC: sedimentation equilibrium analytical ultracentrifugation
SEC-MALLS: size-exclusion chromatography coupled to multiangle laser light scattering
SAXS: small-angle X-ray scattering
sTAM: soluble TAM ECR
SPR: surface plasmon resonance
TIE: tyrosine kinase and immunoglobulin-like and epidermal growth factor homology domains
TM: transmembrane
TRKA: tropomyosin receptor kinase A

## References

[1] Lemke G. (2013) Biology of the TAM receptors. Cold Spring Harb. Perspect. Biol. 5, a009076 24186067 10.1101/cshperspect.a009076 PMC3809585

[2] Rothlin C.V., Carrera-Silva E.A., Bosurgi L. and Ghosh S. (2015) TAM receptor signaling in immune homeostasis. Annu. Rev. Immunol. 33, 355–391 25594431 10.1146/annurev-immunol-032414-112103 PMC4491918

[3] McShane L., Tabas I., Lemke G., Kurowska-Stolarska M. and Maffia P. (2019) TAM receptors in cardiovascular disease. Cardiovasc. Res. 115, 1286–1295 30980657 10.1093/cvr/cvz100 PMC6587925

[4] Graham D.K., DeRyckere D., Davies K.D. and Earp H.S. (2014) The TAM family: phosphatidylserine sensing receptor tyrosine kinases gone awry in cancer. Nat. Rev. Cancer 14, 769–785 25568918 10.1038/nrc3847

[5] Rothlin C.V. and Lemke G. (2010) TAM receptor signaling and autoimmune disease. Curr. Opin. Immunol. 22, 740–746 21030229 10.1016/j.coi.2010.10.001 PMC2997887

[6] Miao Y.R., Rankin E.B. and Giaccia A.J. (2024) Therapeutic targeting of the functionally elusive TAM receptor family. Nat. Rev. Drug Discov. 23, 201–217 38092952 10.1038/s41573-023-00846-8 PMC11335090

[7] Haqshenas G. and Doerig C. (2019) Targeting of host cell receptor tyrosine kinases by intracellular pathogens. Sci. Signal. 12, eaau9894 31530732 10.1126/scisignal.aau9894

[8] Moller-Tank S. and Maury W. (2014) Phosphatidylserine receptors: enhancers of enveloped virus entry and infection. Virology 468-470, 565–580 25277499 10.1016/j.virol.2014.09.009 PMC4252826

[9] Moran S.J. and Starbird C.A. (2026) TAM receptor tyrosine kinases as potential mediators of the non-lytic spread of non-enveloped viruses. Biochem. Soc. Trans. 54, 501–512 42101422 10.1042/BST20250119 PMC13161192

[10] Akalu Y.T., Rothlin C.V. and Ghosh S. (2017) TAM receptor tyrosine kinases as emerging targets of innate immune checkpoint blockade for cancer therapy. Immunol. Rev. 276, 165–177 28258690 10.1111/imr.12522 PMC5381815

[11] Peeters M.J.W., Dulkeviciute D., Draghi A., Ritter C., Rahbech A., Skadborg S.K. et al. (2019) MERTK acts as a costimulatory receptor on human CD8^+^ T cells. Cancer Immunol. Res. 7, 1472–1484 31266785 10.1158/2326-6066.CIR-18-0841

[12] Lemke G. (2017) Phosphatidylserine is the signal for TAM receptors and their ligands. Trends Biochem. Sci 42, 738–748 28734578 10.1016/j.tibs.2017.06.004 PMC5600686

[13] Burstyn-Cohen T. and Fresia R. (2023) TAM receptors in phagocytosis: beyond the mere internalization of particles. Immunol. Rev. 319, 7–26 37596991 10.1111/imr.13267

[14] Lew E.D., Oh J., Burrola P.G., Lax I., Zagorska A., Traves P.G. et al. (2014) Differential TAM receptor-ligand-phospholipid interactions delimit differential TAM bioactivities. eLife 3, e03385 25265470 10.7554/eLife.03385 PMC4206827

[15] Geng K., Kumar S., Kimani S.G., Kholodovych V., Kasikara C., Mizuno K. et al. (2017) Requirement of gamma-carboxyglutamic acid modification and phosphatidylserine binding for the activation of Tyro3, Axl, and Mertk receptors by growth arrest-specific 6. Front. Immunol. 8, 1521 29176978 10.3389/fimmu.2017.01521 PMC5686386

[16] Meyer A.S., Zweemer A.J. and Lauffenburger D.A. (2015) The AXL receptor is a sensor of ligand spatial heterogeneity. Cell Syst. 1, 25–36 26236777 10.1016/j.cels.2015.06.002 PMC4520549

[17] Zhang L., Shim B.S., Kitzmiller C., Kwon Y.C., Richard A.S., Farzan M.R. et al. (2025) Variation in virion phosphatidylserine content drives differential GAS6 binding among closely related flaviviruses. J. Virol. 99, e0111125 40990511 10.1128/jvi.01111-25 PMC12548462

[18] Barton W.A., Dalton A.C., Seegar T.C., Himanen J.P. and Nikolov D.B. (2014) Tie2 and Eph receptor tyrosine kinase activation and signaling. Cold Spring Harb. Perspect. Biol. 6, a009142 24478383 10.1101/cshperspect.a009142 PMC3949358

[19] Pasquale E.B. (2024) Eph receptor signaling complexes in the plasma membrane. Trends Biochem. Sci 49, 1079–1096 39537538 10.1016/j.tibs.2024.10.002 PMC11967910

[20] Leppanen V.M., Saharinen P. and Alitalo K. (2017) Structural basis of Tie2 activation and Tie2/Tie1 heterodimerization. Proc. Natl. Acad. Sci. U.S.A. 114, 4376–4381 28396439 10.1073/pnas.1616166114 PMC5410806

[21] Moore J.O., Lemmon M.A. and Ferguson K.M. (2017) Dimerization of Tie2 mediated by its membrane-proximal FNIII domains. Proc. Natl. Acad. Sci. U.S.A. 114, 4382–4387 28396397 10.1073/pnas.1617800114 PMC5410832

[22] Seiradake E., Harlos K., Sutton G., Aricescu A.R. and Jones E.Y. (2010) An extracellular steric seeding mechanism for Eph-ephrin signaling platform assembly. Nat. Struct. Mol. Biol. 17, 398–402 20228801 10.1038/nsmb.1782 PMC3672960

[23] Xu K., Tzvetkova-Robev D., Xu Y., Goldgur Y., Chan Y.P., Himanen J.P. et al. (2013) Insights into Eph receptor tyrosine kinase activation from crystal structures of the EphA4 ectodomain and its complex with ephrin-A5. Proc. Natl. Acad. Sci. U.S.A. 110, 14634–14639 23959867 10.1073/pnas.1311000110 PMC3767517

[24] Seiradake E., Schaupp A., del Toro Ruiz D., Kaufmann R., Mitakidis N., Harlos K. et al. (2013) Structurally encoded intraclass differences in EphA clusters drive distinct cell responses. Nat. Struct. Mol. Biol. 20, 958–964 23812375 10.1038/nsmb.2617 PMC3941021

[25] Saharinen P., Leppanen V.M. and Alitalo K. (2017) SnapShot: angiopoietins and their functions. Cell 171, 724e721–724e721 10.1016/j.cell.2017.10.009 29053972

[26] Lu Q., Gore M., Zhang Q., Camenisch T., Boast S., Casagranda F. et al. (1999) Tyro-3 family receptors are essential regulators of mammalian spermatogenesis. Nature 398, 723–728 10227296 10.1038/19554

[27] Burstyn-Cohen T., Lew E.D., Traves P.G., Burrola P.G., Hash J.C. and Lemke G. (2012) Genetic dissection of TAM receptor-ligand interaction in retinal pigment epithelial cell phagocytosis. Neuron 76, 1123–1132 23259948 10.1016/j.neuron.2012.10.015 PMC3530147

[28] Ghosh S., Finnemann S.C., Vollrath D. and Rothlin C.V. (2024) In the eyes of the beholder-new Mertk knockout mouse and re-evaluation of phagocytosis versus anti-inflammatory functions of MERTK. Int. J. Mol. Sci. 25, 5299 38791338 10.3390/ijms25105299 PMC11121519

[29] Lemke G. and Rothlin C.V. (2008) Immunobiology of the TAM receptors. Nat. Rev. Immunol. 8, 327–336 18421305 10.1038/nri2303 PMC2856445

[30] Zagorska A., Traves P.G., Lew E.D., Dransfield I. and Lemke G. (2014) Diversification of TAM receptor tyrosine kinase function. Nat. Immunol. 15, 920–928 25194421 10.1038/ni.2986 PMC4169336

[31] Tsou W.I., Nguyen K.Q., Calarese D.A., Garforth S.J., Antes A.L., Smirnov S.V. et al. (2014) Receptor tyrosine kinases, TYRO3, AXL, and MER, demonstrate distinct patterns and complex regulation of ligand-induced activation. J. Biol. Chem. 289, 25750–25763 25074926 10.1074/jbc.M114.569020 PMC4162177

[32] Nagata K., Ohashi K., Nakano T., Arita H., Zong C., Hanafusa H. et al. (1996) Identification of the product of growth arrest-specific gene 6 as a common ligand for Axl, Sky, and Mer receptor tyrosine kinases. J. Biol. Chem. 271, 30022–30027 8939948 10.1074/jbc.271.47.30022

[33] Stitt T.N., Conn G., Gore M., Lai C., Bruno J., Radziejewski C. et al. (1995) The anticoagulation factor protein S and its relative, Gas6, are ligands for the Tyro 3/Axl family of receptor tyrosine kinases. Cell 80, 661–670 7867073 10.1016/0092-8674(95)90520-0

[34] Burstyn-Cohen T., Heeb M.J. and Lemke G. (2009) Lack of protein S in mice causes embryonic lethal coagulopathy and vascular dysgenesis. J. Clin. Invest. 119, 2942–2953 19729839 10.1172/JCI39325 PMC2752078

[35] Jumper J., Evans R., Pritzel A., Green T., Figurnov M., Ronneberger O. et al. (2021) Highly accurate protein structure prediction with AlphaFold. Nature 596, 583–589 34265844 10.1038/s41586-021-03819-2 PMC8371605

[36] Shi X., Lingerak R., Herting C.J., Ge Y., Kim S., Toth P. et al. (2023) Time-resolved live-cell spectroscopy reveals EphA2 multimeric assembly. Science 382, 1042–1050 37972196 10.1126/science.adg5314 PMC11114627

[37] Xu Y., Robev D., Saha N., Wang B., Dalva M.B., Xu K. et al. (2021) The Ephb2 receptor uses homotypic, head-to-tail interactions within its ectodomain as an autoinhibitory control mechanism. Int. J. Mol. Sci. 22, 10473 34638814 10.3390/ijms221910473 PMC8508685

[38] Kelley L.A., Mezulis S., Yates C.M., Wass M.N. and Sternberg M.J. (2015) The Phyre2 web portal for protein modeling, prediction and analysis. Nat. Protoc. 10, 845–858 25950237 10.1038/nprot.2015.053 PMC5298202

[39] Holm L., Laiho A., Toronen P. and Salgado M. (2023) DALI shines a light on remote homologs: one hundred discoveries. Protein Sci. 32, e4519 36419248 10.1002/pro.4519 PMC9793968

[40] Kulahin N., Kristensen O., Rasmussen K.K., Olsen L., Rydberg P., Vestergaard B. et al. (2011) Structural model and trans-interaction of the entire ectodomain of the olfactory cell adhesion molecule. Structure 19, 203–211 21300289 10.1016/j.str.2010.12.014

[41] Winther M., Berezin V. and Walmod P.S. (2012) NCAM2/OCAM/RNCAM: cell adhesion molecule with a role in neuronal compartmentalization. Int. J. Biochem. Cell Biol. 44, 441–446 22155300 10.1016/j.biocel.2011.11.020

[42] Ling L. and Kung H.J. (1995) Mitogenic signals and transforming potential of Nyk, a newly identified neural cell adhesion molecule-related receptor tyrosine kinase. Mol. Cell. Biol. 15, 6582–6592 8524223 10.1128/MCB.15.12.6582 PMC230911

[43] Heiring C., Dahlback B. and Muller Y.A. (2004) Ligand recognition and homophilic interactions in Tyro3: structural insights into the Axl/Tyro3 receptor tyrosine kinase family. J. Biol. Chem. 279, 6952–6958 14623883 10.1074/jbc.M311750200

[44] Linger R.M., Keating A.K., Earp H.S. and Graham D.K. (2008) TAM receptor tyrosine kinases: biologic functions, signaling, and potential therapeutic targeting in human cancer. Adv. Cancer. Res. 100, 35–83 18620092 10.1016/S0065-230X(08)00002-X PMC3133732

[45] Sasaki T., Knyazev P.G., Clout N.J., Cheburkin Y., Gohring W., Ullrich A. et al. (2006) Structural basis for Gas6-Axl signalling. EMBO J. 25, 80–87 16362042 10.1038/sj.emboj.7600912 PMC1356355

[46] Wang J., Chitsaz F., Derbyshire M.K., Gonzales N.R., Gwadz M., Lu S. et al. (2023) The conserved domain database in 2023. Nucleic Acids Res. 51, D384–D388 36477806 10.1093/nar/gkac1096 PMC9825596

[47] Sato M., Tochio N., Koshiba S., Inoue M., Kigawa T., Yokoyama S. et al. (2005) Solution structures of the fn3 domain of human Proto-oncogene tyrosine-protein kinase MER precursor. Protein Data Bank 10.2210/pdb2DBJ/pdb

[48] Gupta R. and Brunak S. (2002) Prediction of glycosylation across the human proteome and the correlation to protein function. Pac. Symp. Biocomput. 7, 310–322 10.1142/9789812799623_0029 11928486

[49] Ferreira I.G., Pucci M., Venturi G., Malagolini N., Chiricolo M. and Dall'Olio F. (2018) Glycosylation as a main regulator of growth and death factor receptors signaling. Int. J. Mol. Sci. 19, 580 29462882 10.3390/ijms19020580 PMC5855802

[50] Porebska N., Pozniak M., Matynia A., Zukowska D., Zakrzewska M., Otlewski J. et al. (2021) Galectins as modulators of receptor tyrosine kinases signaling in health and disease. Cytok. Growth Factor Rev. 60, 89–106 33863623 10.1016/j.cytogfr.2021.03.004

[51] Takahashi M., Hasegawa Y., Gao C., Kuroki Y. and Taniguchi N. (2016) N-glycans of growth factor receptors: their role in receptor function and disease implications. Clin. Sci. (Lond.) 130, 1781–1792 27612953 10.1042/CS20160273

[52] Soderquist A.M. and Carpenter G. (1984) Glycosylation of the epidermal growth factor receptor in A-431 cells. The contribution of carbohydrate to receptor function. J. Biol. Chem. 259, 12586–12594 6092339 10.1016/S0021-9258(18)90787-8

[53] Whitson K.B., Whitson S.R., Red-Brewer M.L., McCoy A.J., Vitali A.A., Walker F. et al. (2005) Functional effects of glycosylation at Asn-579 of the epidermal growth factor receptor. Biochemistry 44, 14920–14931 16274239 10.1021/bi050751j

[54] Chandler K.B., Leon D.R., Meyer R.D., Rahimi N. and Costello C.E. (2017) Site-specific N-glycosylation of endothelial cell receptor tyrosine kinase VEGFR-2. J. Proteome Res. 16, 677–688 27966990 10.1021/acs.jproteome.6b00738 PMC5291786

[55] Rodrigues J.G., Balmana M., Macedo J.A., Pocas J., Fernandes A., de-Freitas-Junior J.C.M. et al. (2018) Glycosylation in cancer: selected roles in tumour progression, immune modulation and metastasis. Cell. Immunol. 333, 46–57 29576316 10.1016/j.cellimm.2018.03.007

[56] Migdall-Wilson J., Bates C., Schlegel J., Brandao L., Linger R.M., DeRyckere D. et al. (2012) Prolonged exposure to a Mer ligand in leukemia: Gas6 favors expression of a partial Mer glycoform and reveals a novel role for Mer in the nucleus. PloS ONE 7, e31635 22363695 10.1371/journal.pone.0031635 PMC3282750

[57] Liu Y., Lan L., Li Y., Lu J., He L., Deng Y. et al. (2022) N-glycosylation stabilizes MerTK and promotes hepatocellular carcinoma tumor growth. Redox Biol. 54, 102366 35728303 10.1016/j.redox.2022.102366 PMC9214875

[58] Shi X. and Jarvis D.L. (2007) Protein N-glycosylation in the baculovirus-insect cell system. Curr. Drug Targets 8, 1116–1125 17979671 10.2174/138945007782151360 PMC3647355

[59] Tsengenes A., Athanasiou C. and Wade R.C. (2026) Glycosylation-modulated conformational diversity in neurotrophin receptors. Biophys. J. 125, 4407–4419 42001238 10.1016/j.bpj.2026.04.017 PMC13507394

[60] Svergun D.I. (1992) Determination of the regularization parameter in indirect-transform methods using perceptual criteria. J. Appl. Crystallogr. 25, 495–503 10.1107/S0021889892001663

[61] Svergun D.I., Barberato C. and Koch M.H.J. (1995) *CRYSOL*—a program to evaluate X-ray solution scattering of biological macromolecules from atomic coordinates. J. Appl. Crystallogr. 28, 768–773 10.1107/S0021889895007047

[62] Franke D. and Svergun D.I. (2009) DAMMIF, a program for rapid ab-initio shape determination in small-angle scattering. J. Appl. Crystallogr. 42, 342–346 27630371 10.1107/S0021889809000338 PMC5023043

[63] Volkov V.V. and Svergun D.I. (2003) Uniqueness of *ab initio* shape determination in small-angle scattering. J. Appl. Cryst. 36, 860–864 10.1107/S0021889803000268 PMC5023043 27630371

[64] Svergun D.I. (1999) Restoring low resolution structure of biological macromolecules from solution scattering using simulated annealing. Biophys. J. 76, 2879–2886 10354416 10.1016/S0006-3495(99)77443-6 PMC1300260

[65] Meng E.C., Goddard T.D., Pettersen E.F., Couch G.S., Pearson Z.J., Morris J.H. et al. (2023) UCSF ChimeraX: tools for structure building and analysis. Protein Sci. 32, e4792 37774136 10.1002/pro.4792 PMC10588335

[66] Tenstad O., Christakou E., Hodneland Nilsson L., Gausdal G., Micklem D., Kursula P. et al. (2024) *In vivo* turnover and biodistribution of soluble AXL: implications for biomarker development. Sci. Rep. 14, 16141 38997436 10.1038/s41598-024-66665-y PMC11245488

[67] Grant O.C., Wentworth D., Holmes S.G., Kandel R., Sehnal D., Wang X. et al. (2025) Generating 3D Models of Carbohydrates with GLYCAM-Web. bioRxiv 2025.05.08.652828, 10.1101/2025.05.08.652828 41872371

[68] Durand D., Vives C., Cannella D., Perez J., Pebay-Peyroula E., Vachette P. et al. (2010) NADPH oxidase activator p67(phox) behaves in solution as a multidomain protein with semi-flexible linkers. J. Struct. Biol. 169, 45–53 19723583 10.1016/j.jsb.2009.08.009

[69] Panjkovich A. and Svergun D.I. (2016) Deciphering conformational transitions of proteins by small angle X-ray scattering and normal mode analysis. Phys. Chem. Chem. Phys. 18, 5707–5719 26611321 10.1039/C5CP04540A

[70] Petoukhov M.V. and Svergun D.I. (2015) Ambiguity assessment of small-angle scattering curves from monodisperse systems. Acta Crystallogr. D. Biol. Crystallogr. 71, 1051–1058 25945570 10.1107/S1399004715002576

[71] Varadi M., Bertoni D., Magana P., Paramval U., Pidruchna I., Radhakrishnan M. et al. (2024) AlphaFold Protein Structure Database in 2024: providing structure coverage for over 214 million protein sequences. Nucleic. Acids. Res. 52, D368–D375 37933859 10.1093/nar/gkad1011 PMC10767828

[72] Dransfield I. and Farnworth S. (2016) Axl and Mer receptor tyrosine kinases: distinct and nonoverlapping roles in inflammation and cancer? Adv. Exp. Med. Biol. 930, 113–132 27558819 10.1007/978-3-319-39406-0_5

[73] Burstyn-Cohen T. and Hochberg A. (2021) TAM signaling in the nervous system. Brain Plast. 7, 33–46 34631419 10.3233/BPL-210125 PMC8461745

[74] Lemmon M.A. and Schlessinger J. (2010) Cell signaling by receptor tyrosine kinases. Cell 141, 1117–1134 20602996 10.1016/j.cell.2010.06.011 PMC2914105

[75] Fujimori T., Grabiec A.M., Kaur M., Bell T.J., Fujino N., Cook P.C. et al. (2015) The Axl receptor tyrosine kinase is a discriminator of macrophage function in the inflamed lung. Mucosal. Immunol. 8, 1021–1030 25603826 10.1038/mi.2014.129 PMC4430298

[76] Mason E.O., Goldgur Y., Robev D., Freywald A., Nikolov D.B. and Himanen J.P. (2021) Structure of the EphB6 receptor ectodomain. PloS One 16, e0247335 33770085 10.1371/journal.pone.0247335 PMC7997048

[77] Park E.C., Thompson A.P., Murphy J.M., Liang L.Y. and Lucet I.S. (2026) The multifaceted roles of receptor tyrosine pseudokinases in cellular signalling. Biochem. Soc. Trans. 54, 727–743 42267535 10.1042/BST20260770 PMC13259870

[78] Hallgren J., Tsirigos K.D., Pedersen M.D., Almagro Armenteros J.J., Marcatili P., Nielsen H. et al. (2022) DeepTMHMM predicts alpha and beta transmembrane proteins using deep neural networks. bioRxiv 2022.2004.2008.487609 10.1101/2022.04.08.487609

[79] Kabsch W. (2010) Integration, scaling, space-group assignment and post-refinement. Acta Crystallogr. D. Biol. Crystallogr. 66, 133–144 20124693 10.1107/S0907444909047374 PMC2815666

[80] Kabsch W. (2010) Xds. Acta Crystallogr. D. Biol. Crystallogr. 66, 125–132 20124692 10.1107/S0907444909047337 PMC2815665

[81] Agirre J., Atanasova M., Bagdonas H., Ballard C.B., Basle A., Beilsten-Edmands J. et al. (2023) The CCP4 suite: integrative software for macromolecular crystallography. Acta Crystallogr. D Struct. Biol. 79, 449–461 37259835 10.1107/S2059798323003595 PMC10233625

[82] McCoy A.J., Grosse-Kunstleve R.W., Adams P.D., Winn M.D., Storoni L.C. and Read R.J. (2007) Phaser crystallographic software. J. Appl. Crystallogr. 40, 658–674 19461840 10.1107/S0021889807021206 PMC2483472

[83] Liebschner D., Afonine P.V., Baker M.L., Bunkoczi G., Chen V.B., Croll T.I. et al. (2019) Macromolecular structure determination using X-rays, neutrons and electrons: recent developments in Phenix. Acta Crystallogr. D Struct. Biol. 75, 861–877 31588918 10.1107/S2059798319011471 PMC6778852

[84] Emsley P. and Cowtan K. (2004) Coot: model-building tools for molecular graphics. Acta Crystallogr. D. Biol. Crystallogr. 60, 2126–2132 15572765 10.1107/S0907444904019158

[85] Emsley P., Lohkamp B., Scott W.G. and Cowtan K. (2010) Features and development of Coot. Acta Crystallogr. D. Biol. Crystallogr. 66, 486–501 20383002 10.1107/S0907444910007493 PMC2852313

[86] Bricogne G., Blanc E., Brandl M., Flensburg C., Keller P., Paciorek W. et al. (2017) BUSTER version 2.10.3, Global Phasing Ltd., Cambridge, U.K.

[87] Joosten R.P., Long F., Murshudov G.N. and Perrakis A. (2014) The PDB_REDO server for macromolecular structure model optimization. IUCrJ 1, 213–220 25075342 10.1107/S2052252514009324 PMC4107921

[88] Emsley P. and Crispin M. (2018) Structural analysis of glycoproteins: building N-linked glycans with Coot. Acta Crystallogr. D Struct. Biol. 74, 256–263 29652253 10.1107/S2059798318005119 PMC5892875

[89] Schrödinger, LLC (2025) The PyMOL Molecular Graphics System. Version 3.1.0 https://pymol.org

[90] Hopkins J.B., Gillilan R.E. and Skou S. (2017) BioXTAS RAW: improvements to a free open-source program for small-angle X-ray scattering data reduction and analysis. J. Appl. Crystallogr. 50, 1545–1553 29021737 10.1107/S1600576717011438 PMC5627684

[91] Manalastas-Cantos K., Konarev P.V., Hajizadeh N.R., Kikhney A.G., Petoukhov M.V., Molodenskiy D.S. et al. (2021) ATSAS 3.0: expanded functionality and new tools for small-angle scattering data analysis. J. Appl. Crystallogr. 54, 343–355 33833657 10.1107/S1600576720013412 PMC7941305

[92] Mirdita M., Schutze K., Moriwaki Y., Heo L., Ovchinnikov S. and Steinegger M. (2022) ColabFold: making protein folding accessible to all. Nat. Methods 19, 679–682 35637307 10.1038/s41592-022-01488-1 PMC9184281

[93] Abramson J., Adler J., Dunger J., Evans R., Green T., Pritzel A. et al. (2024) Accurate structure prediction of biomolecular interactions with AlphaFold 3. Nature 630, 493–500 38718835 10.1038/s41586-024-07487-w PMC11168924

[94] Kirschner K.N., Yongye A.B., Tschampel S.M., Gonzalez-Outeirino J., Daniels C.R., Foley B.L. et al. (2008) GLYCAM06: a generalizable biomolecular force field. Carbohydrates. J. Comput. Chem. 29, 622–655 17849372 10.1002/jcc.20820 PMC4423547

[95] Krissinel E. and Henrick K. (2007) Inference of macromolecular assemblies from crystalline state. J. Mol. Biol. 372, 774–797 17681537 10.1016/j.jmb.2007.05.022

[96] Tria G., Mertens H.D., Kachala M. and Svergun D.I. (2015) Advanced ensemble modelling of flexible macromolecules using X-ray solution scattering. IUCrJ 2, 207–217 25866658 10.1107/S205225251500202X PMC4392415

[97] Zuo Y., Starbird C.A. and Ferguson K.M. (2026) Crystal structure of the extracellular region of the human receptor tyrosine kinase MERTK. Protein Data Bank PDB: 37NT 10.2210/pdb37nt/pdb

