# Supplementary material for: Structural organization of TAM receptor ectodomains
Source: Biochem J. 2026 Oct 8;483(11):2055–73. doi: 10.1042/BCJ20260584 (PMC13660871; doi:10.1042/BCJ20260584)
Supplement: Supplementary file 1 [file BCJ-2026-0584_supp.pdf]

Supplementary Data For

Structural organization of TAM receptor ectodomains

Chrystal A. Starbird[1,2,*,†], Yuhong Zuo[1,2,*], Kahlil Walker[1,2,‡], Steven E. Stayrook[1,2] & Kathryn M. Ferguson[1,2]

[1]Department of Pharmacology, Yale University School of Medicine, New Haven, CT 06520, USA
[2]Cancer Biology Institute, Yale University, West Haven, CT 06516, USA

[*]These authors contributed equally to this work.
[†]Present address: Department of Biochemistry and Biophysics, University of North Carolina School of Medicine, Chapel Hill, NC 27599, USA
[‡]Present address: Koch Institute for Integrative Cancer Research, Massachusetts Institute of Technology, Cambridge, MA 02139, USA

Correspondence: Kathryn M. Ferguson (kathryn.ferguson@yale.edu)

Supplementary Figures and Tables

Supplementary Figure S1: Sequence alignment of TAM ECRs
Supplementary Figure S2: MERTK^{484} electron density maps and crystal packing interactions.
Supplementary Figure S3: Quality and solution properties of sTAMs.
Supplementary Figure S4: AlphaFold2 Models of TAM receptor ECRs.
Supplementary Figure S5: SAXS scattering profiles and model fitting for sTAMs.

Supplementary Table S1: X-ray crystallographic data collection and refinement statistics.
Supplementary Table S2: Details of SAXS data collection and analysis.

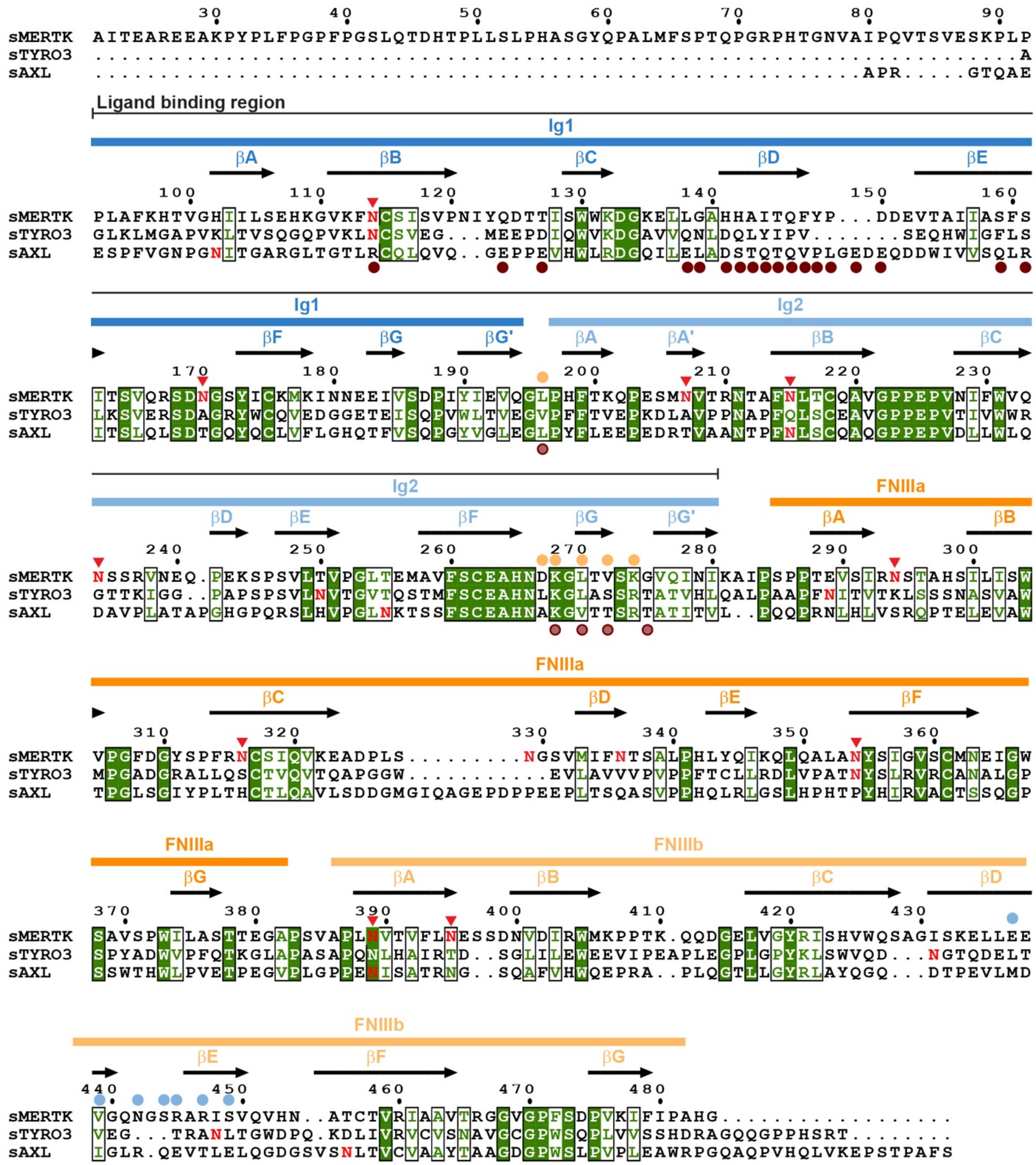

Supplementary Figure S1: Sequence alignment of TAM receptor ectodomains The amino acids in each sTAM were first aligned using Clustal Omega [S1, S2] and the alignment adjusted based on structural alignment of each domain in PyMOL [89]. Color bars above the sequence mark the structural domain, colored blue for Ig1 and Ig2 and orange for FNIIIa and FNIIIa, with the second domain in a lighter shade in each case. The line above denotes the ligand-binding region (Ig1 and Ig2). The positions of the β-strands in the sMERTK crystal structure are shown with arrows and labeled. The amino acid numbering is for the sMERTK sequence. Interface amino acids for which more than 30 Å^2 of their surface area is occluded from solvent upon formation of a complex, evaluated with PDBePISA [95], are indicated by colored circles. Circles

are colored as follows: light orange for amino acids on MERTK Ig2 that are buried by interaction in the crystallographic interaction with FNIIIb', light blue for those on MERTK FNIIIb that are buried by interaction with Ig2', dark red for amino acids buried in site 1 of the GAS6/AXL interaction (Ig1) and lighter red with dark border for those in site 2 (Ig2). The asparagine residues that are glycosylated are shown in red text. A red triangle above the MERTK sequence indicates that at least one sugar residue was seen at this position in the crystal structure. Identical and conserved amino acids are boxed with identity across all three TAM receptors indicated with white text on a green background, and conservation by green text. Pairwise sequence identities across the aligned ECRs are 29%, 30%, and 32% for sMERTK–sTYRO3, sMERTK–sAXL, and sTYRO3–sAXL, respectively; identities across the Ig1–Ig2 ligand-binding region are 34%, 32%, and 35%, respectively. Identity is higher within Ig2 (~35–40%) than within Ig1 (~30%).

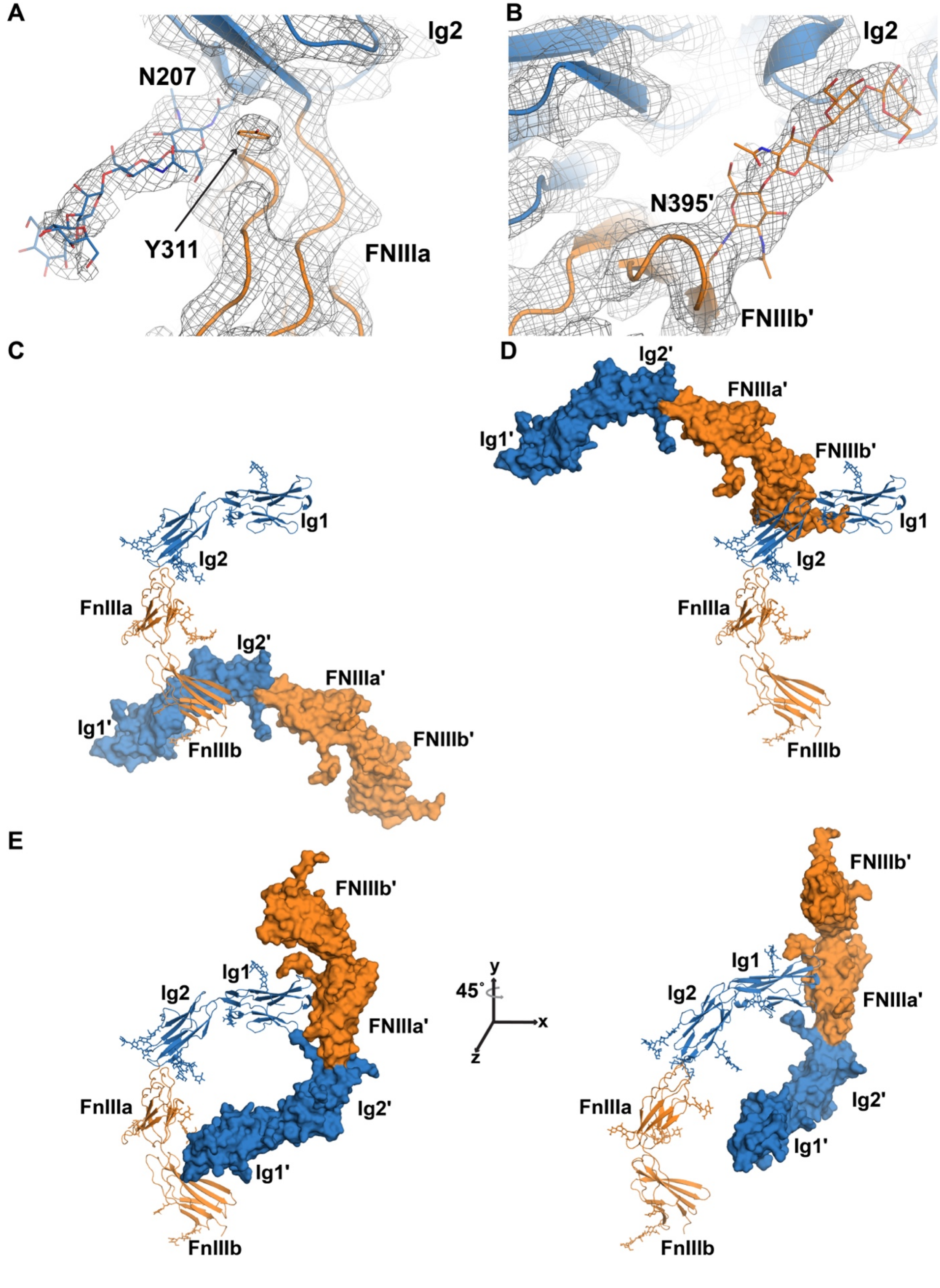

Supplementary Figure S2: MERTK^{484} electron density maps and crystal packing interactions. (A–B) Final refined $2F_o$-F_c electron density map is shown in the region of two

well-ordered glycan chains. The final $MERTK^{484}$ model is shown in cartoon representations colored as in Figure 1, with the glycans shown as sticks, colored by atom type. **(A)** The N207 glycan on Ig2 sits at the Ig2/FNIIIa domain boundary and makes direct contacts with Y311 on FNIIIa. **(B)** The N395 glycan on FNIIIb' makes contacts with the Ig2 domain from a symmetry mate. **(C–D)** Interactions involving Ig2 and FNIIIb are shown, with one MERTK molecule in cartoon representation and the interacting symmetry mate in surface representation. **(C)** Interaction of FNIIIb with Ig2′ of a symmetry mate and **(D)** interaction of Ig2 with FNIIIb′ of a different symmetry mate. **(E)** An additional crystal packing interaction involving Ig1 and FNIIIa' of a second molecule is shown on the left in the same orientation as in C, and rotated 45° about the y-axis on the right.

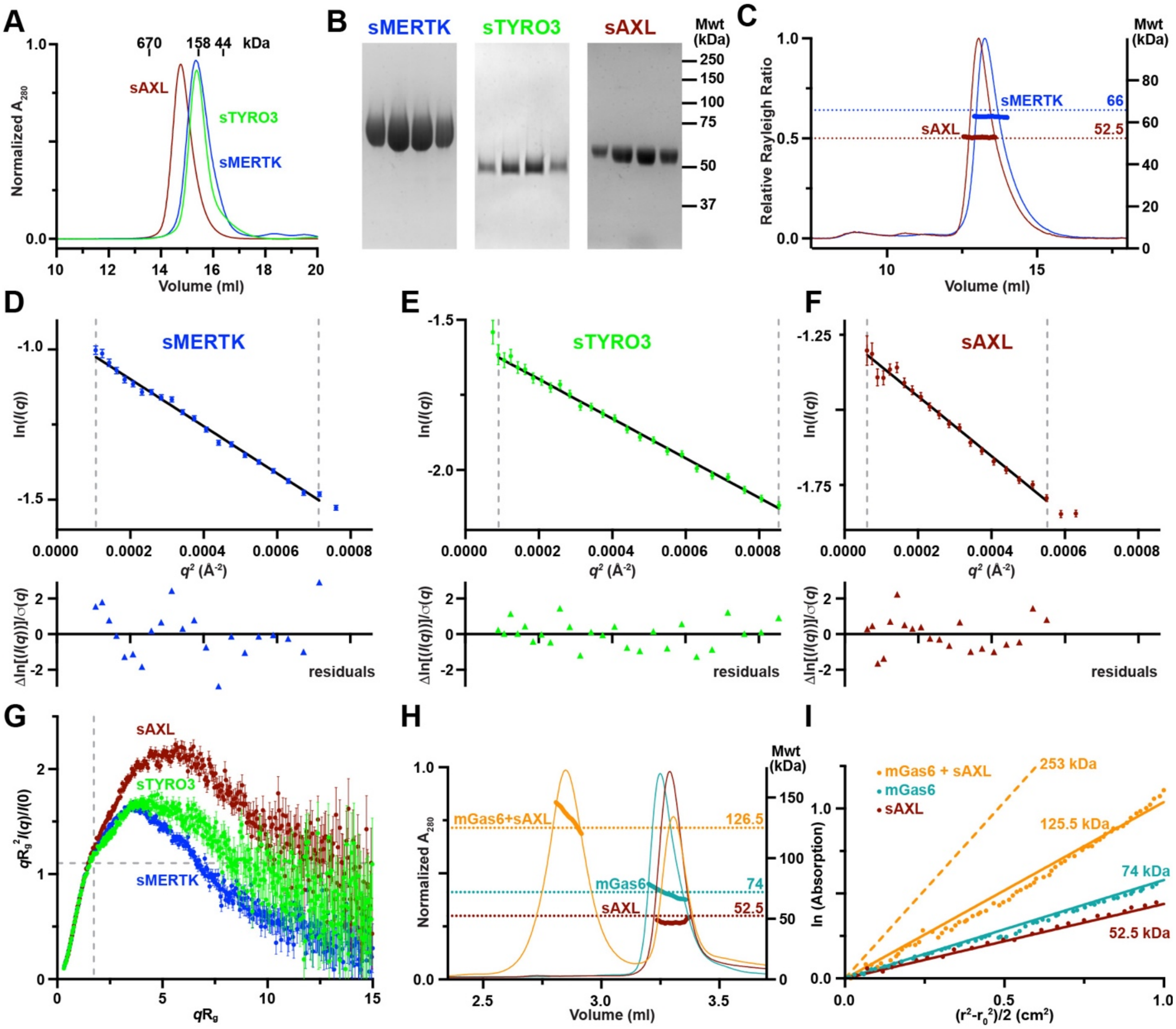

Supplementary Figure S3: Quality and solution properties of sTAMs. (A) Representative chromatograms of sMERTK (blue), sTYRO3 (green) and sAXL (dark red) using Superose 6 SEC column (Cytiva). Normalized absorbance at 280 nm is shown. Positions of elution peaks for globular molecular weight standards are indicated. **(B)** Reducing SDS-PAGE analysis of SEC fractions for each sTAM. Positions of molecular weight standards are shown. **(C)** SEC-MALLS analysis of sMERTK (blue) and sAXL (dark red) loaded at 5.2 and 3.9 mg/mL, respectively, on a Superdex 200 column. Data were collected at BioCAT. Curves show the sTAM concentration as the relative Rayleigh ratio and data points are the weight-average molecular weights calculated from the angular dependence of the scattering as described in the Methods. Dotted lines show the predicted molecular weights for sMERTK and sAXL. **(D–F)** Representative Guinier analysis of home-source SAXS data of **(D)** sMERTK, **(E)** sTYRO3 and **(F)** sAXL. Data where q*R_g < 1.3 are shown, plotted as the natural log of intensity against q^2. The linear variation of the data in this region indicates that the sample is monodisperse. Residuals for the fit are shown below.
(G) Dimensionless Kratky plot of data for sMERTK (blue circles, as shown in Figure 4C), sTYRO3 (green) and sAXL (dark red). Dotted lines are drawn at $(qR_g)^2I(q)/I(0) = 1.104$ and $qR_g = 1.732$ ($3^{1/2}$). Compact globular proteins show a peak at the intersection of these lines. The broader, right-shifted peak for sTAMs is indicative of structural flexibility. A similarly shifted Kratky plot has previously been reported for mouse and human sAXL [66]. **(H)** SEC-MALLS

analysis of sAXL (dark red), mGas6 (teal) and a 1:1 mixture of mGas6 and sAXL, loaded at 30 μM (total protein) using a Zenix-C-SEC300 column collected at Yale. Curves show the protein concentration as UV absorbance at 280 nm and data points are the weight average molecular weights calculated from the angular dependence of the scattering as described in the methods. Dotted lines show the predicted molecular weights for sAXL, mGas6 and a 1:1 complex of sAXL and mGas6. **(I)** Representative SE-AUC data at 8,000 rpm (circular data points) for sAXL (dark red) at 4 μM, mGas6 (teal) at 4 μM and a 1:1 mixture of mGas6 and sAXL (orange) each at 4 μM plotted as in Figure 5E. The lines indicate theoretical slopes for the indicated molecular weights. Solid lines correspond to the predicted monomeric molecular weight for sAXL, mGas6 and a 1:1 complex of the two proteins. The dashed orange line indicates the expected slope if the mGas6:sAXL complex fully dimerizes. Together with the results in J, these data suggest that dimerization of the mGas6:sAXL complex is weak (K_D value estimated > 10 μM). Experiments shown in H and I were performed only once and are consistent with published results [45].

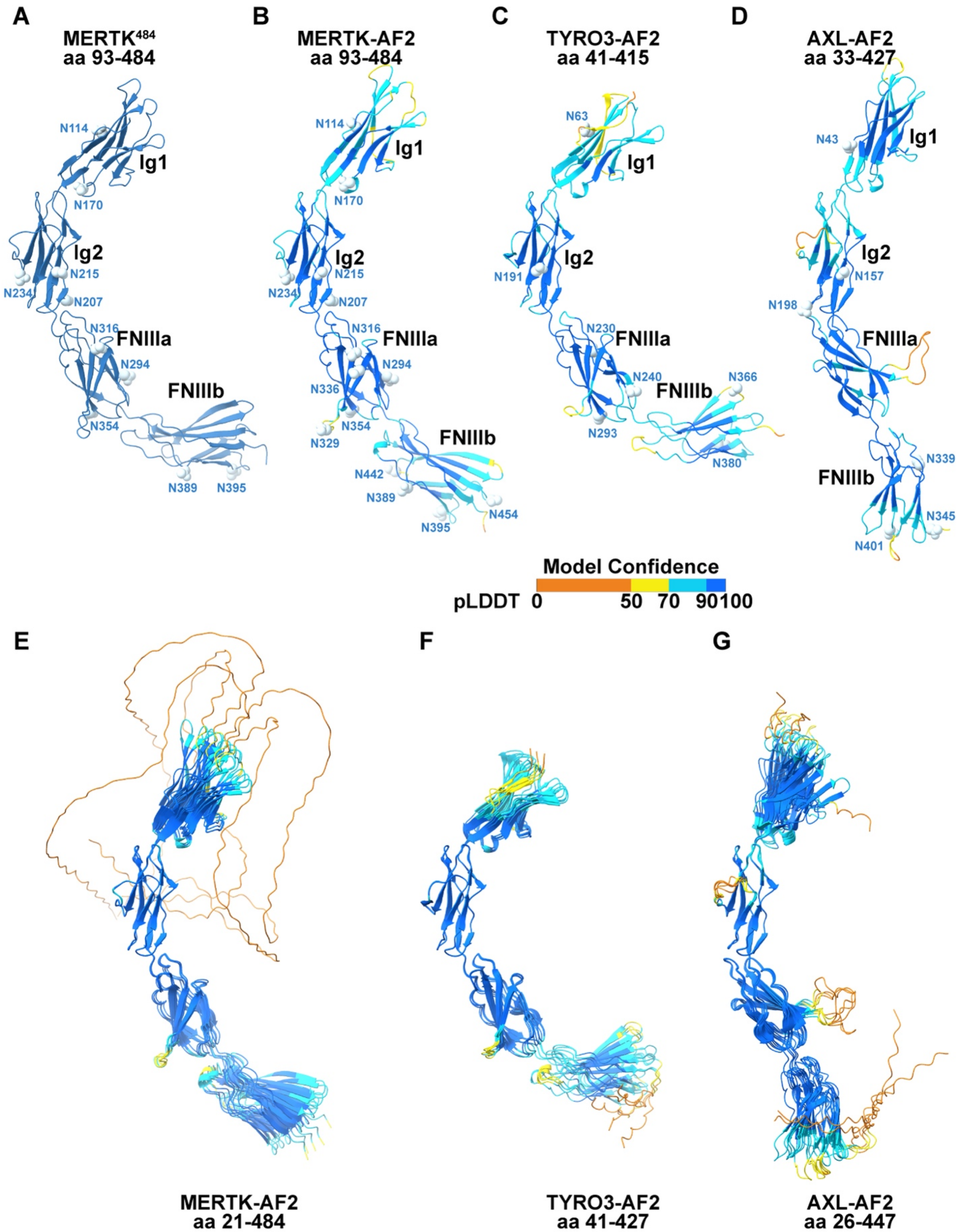

Supplementary Figure S4: AlphaFold2 Models of TAM receptor ECRs. (A) A cartoon representation of the MERTK^{484} structure with the domains labeled and the asparagine side chains at which sugars can be modeled are shown as white spheres and labeled. **(B-D)** Cartoon

representations of the AF2 models of **(B)** MERTK, **(C)** TYRO3, and **(D)** AXL oriented such that the Ig2 is aligned with the Ig2 of $MERTK^{484}$ in A. Structures are colored by model confidence (pLDDT), with all predicted N-linked glycosylation sites indicated as in A. The ranges of amino acids shown here are those used in structural modeling. **(E–G)** A set of five AF2 models for each TAM ECR calculated using ColabFold [92]. Structures are overlaid using Ig2 and colored by pLDDT as in B-D. The range of amino acids shown here corresponds to the predicted range for each sTAM secreted from Sf9 cells using the native signal peptides. The identity of the first amino acid was not confirmed experimentally and here reflects the signal peptide cleavage site from Uniprot. SignalP-6.0 [S3] suggests a different cleavage site for MERTK and AXL – after amino acid 25 for MERTK (probability of 0.74) and after amino acid 32 for AXL (probability 0.98).

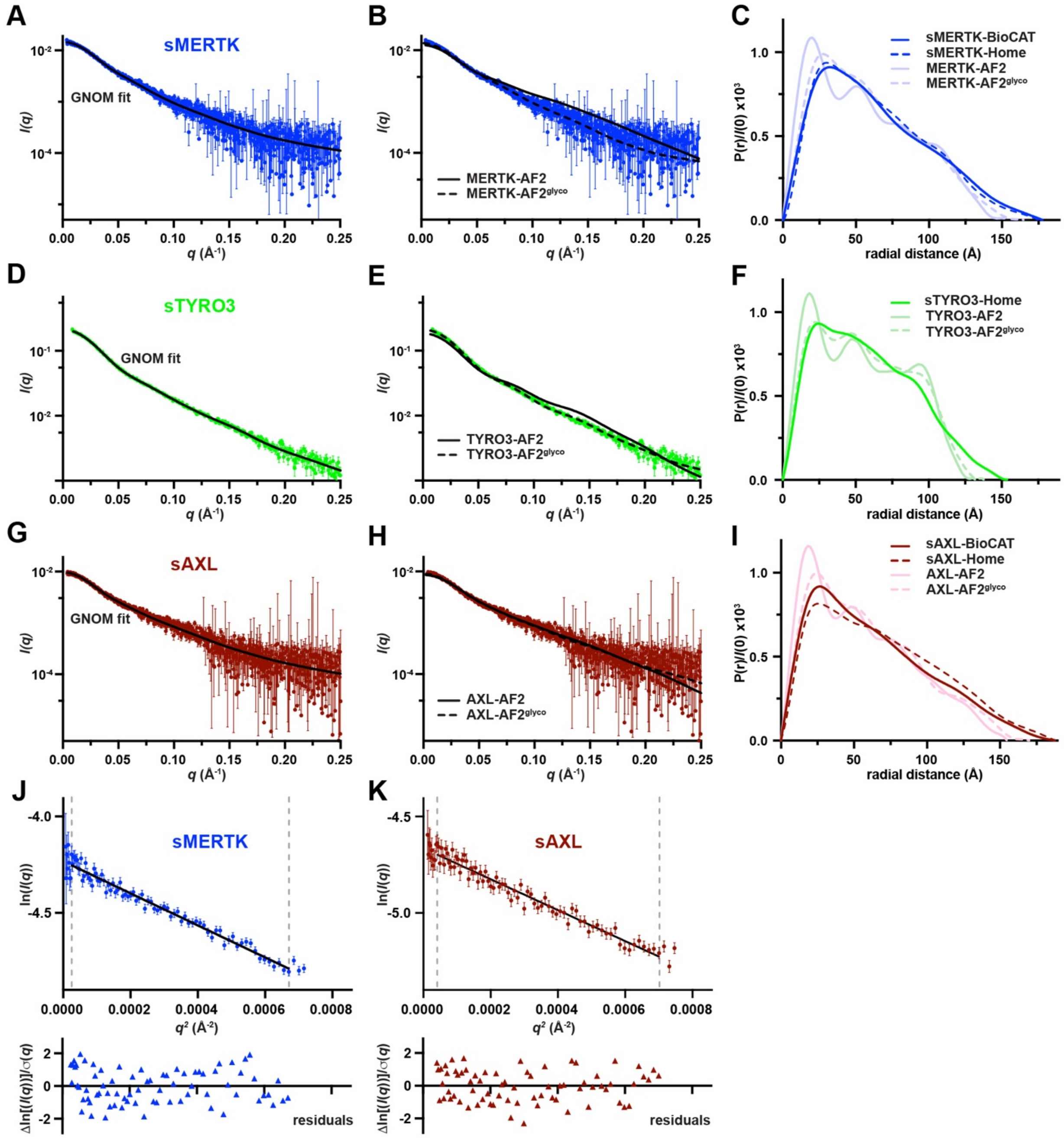

Supplementary Figure S5: SAXS data for sTAMs. (A–I) For each sTAM, SAXS scattering profiles plotted as in Figure 4A are shown with **(A,D,G)** the GNOM [60] fit (black curve) and **(B,E,H)** theoretical scattering profiles calculated using CRYSOL [61] for the relevant AF2 and glycosylated AF2 models (solid and dashed black lines, respectively). **(C,F,I)** The pair-distance distribution (P(r)) curves calculated from the sTAM scattering profiles are shown as solid, dark-colored lines. The P(r) curves derived from the CRYSOL-generated scattering profiles are shown in lighter colors, with solid lines for the AF2 models and dashed lines for the glycosylated AF2 models. **(A–C)** Data for sMERTK from SEC-SAXS at BioCAT, loaded at 5.2 mg/mL (blue data points and curves). **(D–F)** Data for sTYRO3 at 3.4 mg/mL from the home source (green data points and curves). **(G–I)** Data for sAXL from SEC-SAXS at BioCAT, loaded at 3.8 mg/mL (dark red data points and curves). **(J–K)** Guinier analysis for sMERTK and sAXL BioCAT data, plotted as in Supplementary Figure S3D–E and colored as in A and G. See Methods for details.

Supplementary Table S1: X-ray crystallographic data collection and refinement statistics.

	**sMERTK**<br>PDB:37NT
**Data Collection Statistics**	
X-ray source	APS 23-ID-D
Wavelength (Å)	1.0332
Space group	$P2_12_12_1$
Unique cell dimensions	a = 40.5 Å b = 133.5 Å c = 163.8 Å
Resolution Range (Å)[a]	50.0 – 3.4 (3.63 – 3.4)
Total/Unique Reflections	64141 / 12712
Fold Redundancy[a]	5.0 (5.2)
Completeness (%)[a]	99.3 (97.0)
R_{meas}[a]	0.15 (3.7)
<I/σ>[a]	7.6 (0.39)
$CC^{1/2}$ (last shell)	0.17
Wilson B-factor (Å)	136.5
**Refinement Statistics**	
Resolution range (Å)	43.0 – 3.41
# reflections / # test set	12618 / 618
R factor / R_{free}	0.256 / 0.307
Model	One MERTK ECR molecule
Total number of atoms	3435
Water Molecules	-
Average B-factors (Å^2)	155
RMSD bond lengths (Å)	0.002
RMSD bond angles (º)	0.648
Ramachandran Favored (%)	94.10
Allowed	5.13
Outliers	0.77

[a]Values in parentheses refer to the highest resolution shell.

Supplementary Table S2: Details of SAXS data collection and analysis.

**SAXS data-collection parameters**					
Instrument	Rigaku BioSAXS-2000nano 2D Kratky block camera system with Rigaku HyPix-3000 HPAD CCD detector			BioCAT, Sector 18, with Pilatus3 X IM (Dectris) detector	
Source	Rigaku 007HF rotating anode			Advanced Photon Source	
Wavelength (Å)	1.5406			1.033	
Camera Length (m)	0.49			3.651	
Abs. scaling	Comparison to scattering with pure H_2O			1.055 mm glassy carbon	
Exposure Time (s)	7200			0.5 every 1 s	
Sample configuration	Quartz glass capillary			SEC-MALS-SAXS	
Sample temp. (°C)	20			22	
q-meas. range ($Å^{-1}$)	0.007-0.5			0.0021-0.34	
**Sample:**	sMERTK	sTYRO3	sAXL	sMERTK	sAXL
Organism	*H. sapiens*	*H. sapiens*	*H. sapiens*	*H. sapiens*	*H. sapiens*
Source	Sf9	Sf9	Sf9	Sf9	Sf9
Sequence ID	Q12866	Q06418	P30530	Q12866	P30530
(amino acids)	(21-484)	(41-427)	(26-447)	(21-484)	(26-447)
ε 280 nm ($M^{-1}cm^{-1}$)	59275	79450	61795	59275	61795
Predicted # N-glycans	12	7	6	12	6
Estimated mass (kDa)[1]	65.9	49.6	52.4	65.9	52.4
Conc (mg/ml)	1.8-13.0	1.75-9.0	1.9-7.4	-	-
SEC load conc (mg/ml)	-	-	-	5.2	3.8
**Structural parameters[1]:**					
Conc (mg/ml)	3.4	2.6	3.6	-	-
Guinier Analysis					
$I(0)$ (cm^{-1})	0.39 ± 0.002	0.21 ± 0.001	0.28 ± 0.002	-	-
$I(0)$ (arb. unit)	-	-	-	0.01 ± 8 x 10^{-5}	0.009 ± 7 x 10^{-5}
$I(0)/c$ (arb. unit)	0.115	0.082	0.079	-	-
R_g (Å)	48.5 ± 0.3	44.4 ± 0.4	54.6 ± 0.7	49.9 ± 0.5	49.1 ± 0.6
q-range ($Å^{-1}$)	0.0103-0.0267	0.0095-0.0292	0.0078-0.0235	0.005-0.0259	0.0064-0.0265
qR_g max	1.298	1.299	1.28	1.293	1.316
R^2 (corr. coefficient)	0.992	0.996	0.986	0.973	0.960
Mwt from $I(0)$ (kDa)	72.4	51.8	51.3	-	-
P(r) analysis					
$I(0)$ (cm^{-1})	0.39 ± 0.002	0.21 ± 0.001	0.28 ± 0.002	0.01 ± 8 x 10^{-5}	0.01 ± 8 x 10^{-5}
R_g (Å)	50.9 ± 0.3	46.8 ± 0.4	55.9 ± 0.5	51.8 ± 0.4	52.6 ± 0.7
D_{max} (Å)	175	154	187	178	188
q-range for fits ($Å^{-1}$)	0.0111-0.2383	0.0095-0.2506	0.0152-0.290	0.0041-0.268	0.0041-0.288
χ^2 (GNOM total est.)	0.998 (0.751)	0.93 (0.806)	1.025 (0.715)	1.071 (0.754)	0.980 (0.712)
Quality of solution	GOOD	GOOD	REASONABLE	GOOD	REASONABLE
Ambiguity score	2.63	2.51	2.24	2.51	2.00

Shape model-fitting results[2]

Sample:	sMERTK	sTYRO3	sAXL	sMERTK	sAXL
Instrument	Rigaku BioSAXS			BioCAT	
Representative DAMMIF results, 40 calculations					
q-range for fits ($Å^{-1}$)	0.0111-0.1650	0.0095-0.1799	0.0152-0.1444	0.0041-0.1601	0.0041-0.1629
Symmetry	P1	P1	P1	P1	P1
Anisotropy assumption	None	None	None	None	None
NSD	0.85 ± 0.15	1.16 ± 0.24	1.15 ± 0.48	1.02 ± 0.17	1.10 ± 0.37
# clusters (excluded)	8 (0)	3 (3)	9 (1)	11 (4)	10 (0)
Ensemble Res. (Å)	37	42	41	46	39
χ^2	1.121 ± 0.005	0.913 ± 0.002	1.006 ± 0.008	1.285 ± 0.002	1.143 ± 0.001
R_g (Å)	50.91 ± 0.02	46.81 ± 0.02	57.60 ± 0.02	52 ± 0	52 ± 0
D_{max} (Å)	187 ± 3	166 ± 2	208 ± 4	186 ± 3	187 ± 4
Refined χ^2	1.16	0.91	1.03	1.29	1.13
Refine R_g (Å)	51.3	47.2	57.8	53.7	52.2
Refine D_{max} (Å)	174	166	196	177	184

Software employed for SAXS data reduction, analysis and interpretation

Data reduction & processing	BioXTAS RAW	Version 2.4.0
Extinction coefficient	ProtParam	https://web.expasy.org/protparam/
Shape/bead modeling	DAMMIF *via* RAW	ATSAS version 4.1.2-2-20250926
Additional analysis	ASTAS online	Release 3.2.2

Fitting of models to scattering data using CRYSOL

Sample:	sMERTK				sTYRO3		sAXL	
Model	$MERTK^{484}$	$MERTK^{glyco}$	AF2	$AF2^{glyco}$	AF2	$AF2^{glyco}$	AF2	$AF2^{glyco}$
GNOM R_g (Å)	45.3	45.8	46.5	48.0	44.2	45.3	46.3	48.7
D_{max} (Å)	152	155	152	165	132	138	155	172
χ^2 values								
Home data	17	2.4	55	1.7	14.5	1.2	5.5	1.3
BioCAT data	1.7	1.3	4.8	1.2	-	-	1.2	1.0

1. Data here are for a representative dataset with errors indicating the error of the fits. Mean values over biological replicates for key measurements are listed in Table 1.
2. Mean and standard deviation for the included bead models.

Supplementary References

S1. Sievers, F., Wilm, A., Dineen, D., Gibson, T. J., Karplus, K., Li, W., et al. (2011) Fast, scalable generation of high-quality protein multiple sequence alignments using Clustal Omega. *Mol. Syst. Biol.* **7**, 539 https://doi.org/10.1038/msb.2011.75

S2. Madeira, F., Madhusoodanan, N., Lee, J., Eusebi, A., Niewielska, A., Tivey, A. R. N., et al. (2024) The EMBL-EBI Job Dispatcher sequence analysis tools framework in 2024. *Nucleic Acids Res.* **52**, W521–W525 https://doi.org/10.1093/nar/gkae241

S3. Teufel, F., Almagro Armenteros, J. J., Johansen, A. R., Gislason, M. H., Pihl, S. I., Tsirigos, K. D., et al. (2022) SignalP 6.0 predicts all five types of signal peptides using protein language models. *Nat. Biotechnol.* **40**, 1023–1025 https://doi.org/10.1038/s41587-021-01156-3
